# Mechanistic and Kinetic Studies on the Homogeneous Gas-Phase Formation of PCTA/DTs from 2,4-Dichlorothiophenol and 2,4,6-Trichlorothiophenol

**DOI:** 10.3390/ijms160920449

**Published:** 2015-08-28

**Authors:** Fei Xu, Xiangli Shi, Yunfeng Li, Qingzhu Zhang

**Affiliations:** Environment Research Institute, Shandong University, Jinan 250100, China; E-Mails: xufei@sdu.edu.cn (F.X.); 123songchuan@163.com (X.S.); liyfeng1028@gmail.com (Y.L.)

**Keywords:** 2,4-dithiochlorophenol, 2,4,6-Trithiochlorophenol, PCTA/DT, formation mechanism, rate constants

## Abstract

Polychlorinated thianthrene/dibenzothiophenes (PCTA/DTs) are sulfur analogues compounds to polychlorinated dibenzo-p-dioxin/dibenzofurans (PCDD/Fs). Chlorothiophenols (CTPs) are key precursors to form PCTA/DTs. 2,4-DCTP has the minimum number of Cl atoms to form 2,4,6,8-tetrachlorinated dibenzothiophenes (2,4,6,8-TeCDT), which is the most important and widely detected of the PCDTs. In this paper, quantum chemical calculations were carried out to investigate the homogeneous gas-phase formation of PCTA/DTs from 2,4-DCTP and 2,4,6-TCTP precursors at the MPWB1K/6-311+G(3df,2p)//MPWB1K/6-31+G(d,p) level. Several energetically feasible pathways were revealed to compare the formation potential of PCTA/DT products. The rate constants of the crucial elementary reactions were evaluated by the canonical variational transition-state (CVT) theory with the small curvature tunneling (SCT) correction over a wide temperature range of 600–1200 K. This study shows that pathways that ended with elimination of Cl step were dominant over pathways ended with elimination of the H step. The water molecule has a negative catalytic effect on the H-shift step and hinders the formation of PCDTs from 2,4-DCTP. This study, together with works already published from our group, clearly illustrates an increased propensity for the dioxin formation from CTPs over the analogous CPs.

## 1. Introduction

Polychlorinated thianthrene/dibenzothiophenes (PCTA/DTs) are sulfur-containing compounds and have environmentally and toxicologically attracted interest from public and regulatory concerns, due to their resemblance of geochemical behavior, toxicity and physicochemical properties in the environment as polychlorinated dibenzo-p-dioxin/dibenzofurans (PCDD/Fs) [1,2,3,4,5,6,7,8,9]. PCTA/DTs are PCDD/Fs analogues in which the oxygen atoms are substituted by the sulfur atoms. PCDT/TAs were never intentionally synthesized for commercial purposes, but are known to be produced as byproducts from the chemical processes that are similar to those resulting in the formation of PCDD/Fs, such as combustion, emissions from municipal and hazardous waste incinerators as well as industrial incinerators [10,11]. Buser reported the concentrations of PCDTs in fly ash was to be up to 55 ng/g, at or 1 magnitude below the concentrations of the PCDDs and PCDFs [10]. In iron and steel sintering plants, the presence of PCDTs in emission was found at approximately the same concentrations as PCDFs [12]. Thus, considering the high toxicity and wide distribution, clarifying PCTA/DT formation and emission from combustion and thermal processes have important significant in preventing the dangers of PCTA/DTs in the environment and improving both municipal and hazardous waste incinerators.

The most direct route to the formation of PCTA/DTs is the gas-phase reaction of chemical precursors. Chlorophenols (CPs) are key precursors in essentially all proposed pathways of the formation of PCDD/Fs [13,14,15,16,17,18]. Chlorothiophenols (CTPs) are structurally similar to CPs and have been demonstrated to be the predominant precursors or key intermediates of PCTA/DT formation [19,20,21]. CTPs have been widely employed as intermediates in large quantities in various chemical industries, such as in the manufacturing of dyes, insecticides, printing inks, pharmaceuticals and polyvinyl chloride [19]. In particular, the dichlorothiophenols (DCTPs) are used in pharmaceutical industry due to their inhibitory effect on human cytochrome [22]. CTPs are toxic and hazardous to human health and the environment due to the presence of sulfur and chlorine [23]. Several studies shows that the toxicity of CTPs are influenced by chlorine substitution number and substitution pattern [23,24], and the order of toxicity of CTPs increases with the degree of chlorination [23,24]. The understanding of the reaction mechanism is crucial for any attempt to prevent PCTA/DTs. The high correlation between the concentrations of PCTA/DTs and PCDD/Fs in the environmental samples indicated the formation of PCTA/DTs involve analogous formation steps to the mechanism of formation of PCDD/Fs [25,26,27]. Under the pyrolysis or combustion conditions, CTPs can cause loss of the thiophenoxyl-hydrogen to form chlorothiophenoxy radicals (CTPRs). Similar to the formation of PCDD/Fs from CP precursors, the gas-phase formation of PCTA/DTs from CTP precursors was also proposed involving radical-radical coupling of two CTPRs and radical-molecule recombination of CTPR and CTP. The recent works have shown that radical-radical coupling are more competitive thermodynamically than radical-molecule recombination for the PCTA/DT formation [13,14,20,21,28,29,30]. The dimerization of CTPRs is the major PCDT/TA formation pathway [20,21].

In this paper, we deeply investigate the homogeneous gas-phase formation of PCTA/DTs from 2,4-dichlorothiophenol (2,4-DCTP) and 2,4,6-trichlorothiophenol (2,4,6-TCTP) as precursors, using a direct density functional theory (DFT) kinetic study. In recent researches from this laboratory, we investigated the formation of PCDD/Fs from 2,4-dichlorophenol and 2,4,6-trichlorophenol as precursors [30]. Thus, as part of our ongoing work in the field, it is important to compare the formation of PCTA/DTs from CTPs and the formation of PCDD/Fs from CPs. The rate constants of the elementary reactions involved in the PCTA/Fs formations from 2,4-DCTP and 2,4,6-TCTP were evaluated over a wide temperature range of 600–1200 K. The effect of oxygen atom of PCDD/Fs substituted by sulfur atoms on the formation mechanisms and the influence of water molecular on the formation potential of PCTA/DTs are discussed.

The reasons for initiating such a work are as follows. First, due to the high toxicity of PCTA/DTs and the lack of efficient detection schemes for intermediate radicals, the specific formation mechanism of PCTA/DTs remains unclear. Quantum chemical calculation is a useful method to establish the pathway feasibility and confirm the product priority, especially for the highly toxic compounds. Over the last few decades, numerous effect have been undertaken to investigate the PCDD/Fs formation from CPs. However, corresponding research pertinent to the PCTA/DTs are limited, especially for the theoretical studies. Only two ab initio studies are on record for the formation mechanism of PCTA/DTs from CTPs [20,21]. However, the formation pathways of PDTA via smiles rearrangement steps and the formation pathways of PCDT via double H-shift step followed with H abstraction steps, which were recently suggested to be the major pathway in the homogeneous gas-phase formation of PCDD/Fs from CP precursors [28,29,30], was not investigated in this ab initio study [20,21]. Second, owning to the difference of chlorine substitution positions and numbers, PCTA/DTs are found as mixtures of 75 PCTA isomers and 135 TCDT isomers. Among them, the tetrachlorinated thianthrene/dibenzothiophenes (TeCTA/DTs) are the most widely detected [8,11,25,31,32]. In particular, 2,4,6,8-TeCDT is the most important TeCDT, which was detected not only in river and marine organisms and sediment [4,8,10,33,34], but also in thermal process, municipal solid waste incinerator fly ash samples, pulp and paper mill effluent and metal reclamation industry [10,11,12,32,35]. For example, in the sediment of Passaic River and Newark Bay Estuary, the ratio of the 2,4,6,8-TeCDT to 2,3,7,8-TeCDD ratio was approximately 6:1 [33,34]. In crab tissues from the same area, the concentration of 2,4,6,8-TeCDT was five to ten times higher than that of 2,3,7,8-TeCDD [10]. Busher found three most prominent TeCDT isomers, 2,4,6,8-TeCDT, 2,3,6,7-TeCDT and 2,3,7,8-TeCDT, in municipal solid waste incineration fly ash [10]. 2,4-DCTP has the minimum number of Cl atoms to form 2,4,6,8-TeCDT. As a comparison of a previous study of PCDD/Fs formation from 2,4,6-TCP [30], we also invested the PCTA/DT formation from 2,4,6-TCTP, which produced two kind of important TeCTAs, 1,3,7,9-TeCTA and 1,3,6,8-TeCTA. Third, due to the ability to form hydrogen bonds and the abundance and unique properties, water has long been considered an important subject in chemical reactions. For example, the water molecule can promote the nucleation of aerosols by forming hydrogen bonded complexes with many atmospheric species, such as H_2_SO_4_∙H_2_O, NH_3_∙H_2_O, NH(CH_3_)_2_∙H_2_O, HNO_3_∙H_2_O, OH∙H_2_O and HO_2_∙H_2_O [36,37]. In addition, it can participate actively in the gas-phase reaction of Nitro-PAHs (polycyclic aromatic hydrocarbons) arising from the OH^−^ initiated and NO_3_^−^ initiated atmospheric reactions of PAHs [38]. Water vapor is always amply present in industrial operations. In some plants, flue gases are quenched with water before their cleaning. Therefore, the influence of water on the formation of PCTA/DTs needs to be studied. Fourth, in the environmental field, the kinetic models are used to account for the potential outcomes of PCTA/DTs to the environment and the gaseous route in the production of PCTA/DTs in combustion and thermal processes. However, owning to the limitation of experimental conditions and lack of the effect detection methods, the kinetic parameters, such as the pre-exponential factors, the activation energies and the rate constants, of the elementary reactions is insufficiency. This causes difficulties to further improve and optimize PCTA/DTs formation models. In such a situation, an alternative method is to use the calculated rate constant or other dynamical information directly from quantum calculation of electronic structure, frequency and energy.

## 2. Results and Discussion

### 2.1. Formation of 2,4-DCTPRs and 2,4,6-TCTPRs

The formation of CTPRs from CTPs is the initial and key step in the formation of PCTA/DTs. In combustion and thermal processes, CTPRs can be produced through loss of the thiophenoxyl-hydrogen via unimolecular cleavage of the S-H bond or abstracted by the active radicals H, OH, O (^3^P), and Cl. The potential barriers (Δ*E*) and the reaction heats (Δ*H*) of 2,4-DCTP and 2,4,6-TCTP thiophenoxyl-hydrogen cleavage and abstraction by H, OH, O(^3^P), and Cl were calculated at the MPWB1K/6-311+G(3df,2p) level in Table 1. Data of 2,4-DCTP and 2,4,6-TCTP thiophenoxyl-hydrogen cleavage and abstraction by H and OH were cited from our previous studies [39]. All the abstraction steps are strongly exothermic. The potential energy surface scan of S–H bond unimolecular cleavage by varying the S–H bond length shows that there is no transition state in the decomposition process.

**Table 1:** The potential barriers Δ*E* (in kcal/mol) and the reaction heats Δ*H* (in kcal/mol) of 2,4-DCTP and 2,4,6-TCTP thiophenoxyl-hydrogen cleavage and abstraction by H, OH, O(^3^P), and Cl at the MPWB1K/6-311+G(3df,2p) level. Δ*H* is calculated at 0 K.

Reaction	Δ*E*	Δ*H*	Reference
2,4-DCTP → 2,4-DCTPR + H	-	79.15	[39]
2,4-DCTP + H → 2,4-DCTPR + H_2_	3.44	−21.52	[39]
2,4-DCTP + OH → 2,4-DCTPR + H_2_O	8.80	−35.05	[39]
2,4-DCTP + O(^3^P) → 2,4-DCTPR + OH	2.55	−19.33	this paper
2,4-DCTP + Cl → 2,4-DCTPR + HCl	−8.03	−22.94	this paper
2,4,6-TCTP → 2,4,6-TCTPR + H	-	80.55	[39]
2,4,6-TCTP + H → 2,4,6-TCTPR + H_2_	4.27	−20.12	[39]
2,4,6-TCTP + OH → 2,4,6-TCTPR + H_2_O	9.95	−33.65	[39]
2,4,6-TCTP + O(^3^P) → 2,4,6-TCTPR + OH	3.62	−18.08	this paper
2,4,6-TCTP + Cl → 2,4,6-TCTPR + HCl	−6.87	−21.70	this paper

To compare the physical insight of CTPR and CPR, the Mulliken charge and SOMO-LUMO gap of 2,4-DCTPR, 2,4-DCPR, 2,4,6-TCTPR and 2,4,6-TCPR were calculated at MPWB1K/6-31+G(d,p) level. The Mulliken charge of O in 2,4-DCPR (−0.423e) is more negative than that of S in 2,4-DCTPR (−0.182e); The Mulliken charge of O in 2,4,6-TCPR (−0.398e) is more negative than that of S in 2,4,6-TCTPRs (−0.183e). This means the O atom in 2,4-DCPR and 2,4,6-TCPR have stronger nucleophilicity than the S atom in 2,4-DCTPR and 2,4,6-TCTPR. In addition, the SOMO-LUMO gap of 2,4-DCTPR (0.25709 a.u.) and 2,4,6-TCTPR (0.25456 a.u.) is larger than that of 2,4-DCPR (0.25354 a.u.) and 2,4,6-TCPR (0.24785 a.u.), respectively, which indicate that 2,4-DCTPR and 2,4,6-TCTPR are more stable than 2,4-DCPR and 2,4,6-TCPR. Figure S1 depicts the electron density of 2,4-DCTPR, 2,4-DCPR, 2,4,6-TCTPR 2,4,6-TCPR at MPWB1K/6-31+G(d,p) level.

The imaginary frequencies, the zero-point energies and the total energies for the transition states involved in the formation of PCTA/DTs from the 2,4-DCTP and 2,4,6-TCTP as precursors are shown in Table S1. Cartesian coordinates for the reactants, intermediates, transition states and products involved in formation of PCTA/DTs from the 2,4-DCTP and 2,4,6-TCTP as precursors are depicted in Tables S2 and S3.

### 2.2. Formation of PCTAs from 2,4-DCTPRs and 2,4,6-TCTPRs

**Figure 1:**
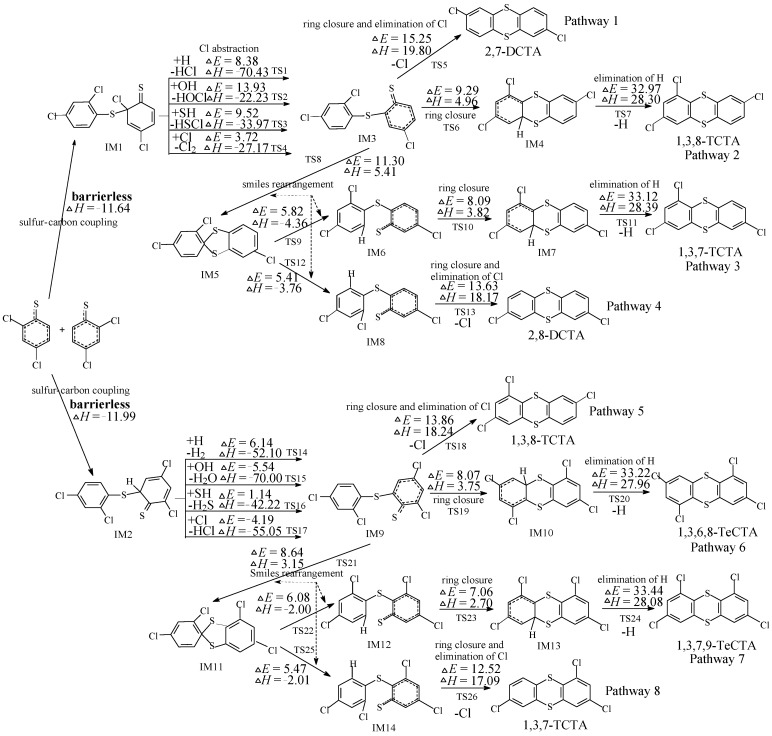
Polychlorinated thianthrene (PCTA) formation routes embedded with the potential barriers Δ*E* (in kcal/mol) and reaction heats Δ*H* (in kcal/mol) from dimerization of 2,4-DCTPRs. Δ*H* is calculated at 0 K.

Figure 1 shows the homogeneous gas-phase formation of PCTAs from 2,4-DCTPR. The potential barriers Δ*E* (in kcal/mol) and reaction heats Δ*H* (in kcal/mol) are calculated at the MPWB1K/6-311+G(3df,2p)//MPWB1K/6-31+G(d,p) level. From Figure 1, eight PCTA formation pathways (pathways 1-8) are proposed from dimerization of 2,4-DCTPRs. All the pathways start with sulfur-carbon coupling step which is barrierless, resulting in two *o*-thiophenoxy-thiophenol (TPOTP) intermediate IM1 (with reaction heat −11.64 kcal/mol) and IM2 (with reaction heat −11.99 kcal/mol).

It can be seen from Figure 1 that pathway 1 and pathway 5 are similar, involving three elementary steps: (1) sulfur-carbon coupling; (2) Cl or H abstraction and (3) ring closure and intra-annular elimination of Cl (They occur in a one-step reaction and are the concerted reaction). The oxygen-carbon coupling of 2,4-DCPRs appears to be barrierless and strongly exothermic. The Cl atom can be abstracted by H, OH, SH and Cl radicals and the Cl abstraction step is highly exothermic. The ring closure and intra-annular elimination of Cl occur in a one-step reaction are the concerted reactions through the transition state TS5 and TS18. This elementary process requires a high potential barrier and is strongly endoergic, and it is the rate-determining step for pathway 1 and pathway 4. From Figure 1, pathways 2 and 6 are analogous, and they contain four elementary pathways: (1) sulfur-carbon coupling; (2) Cl or H abstraction; (3) ring closure and (4) intra-annular elimination of H. The ring closure and elimination of H steps occur separately, and the rate-determining step is the elimination of H. Pathways 3, 4, 7 and 8 contain two smiles rearrangement steps before ring closure step. Pathways 3 and 7 are analogical, and they involve six elementary steps: (1) sulfur-carbon coupling; (2) Cl/H abstraction; (3,4) smiles rearrangement (two elementary steps); (5) ring closure; and (6) intra-annular elimination of H (the rate-determining step). Pathway 4 is similar to pathway 8, which covers five elementary steps: (1) sulfur-carbon coupling; (2) Cl/H abstraction; (3,4) smiles rearrangement (two elementary steps); (5) ring closure and intra-annular elimination of H (the rate-determining step).

Evidently, pathway 1 involves relative less elementary steps compared to pathway 2 and pathway 3, respectively. In addition, the rate determining step involved in pathway 1 has a lower potential barrier and is less endoergic than that involved in pathway 2 and pathway 3, respectively. Therefore, pathway 1 is favored over pathway 2 and pathway 3, respectively. Similarly, pathway 5 is preferred over pathway 6 and pathway 7, respectively. Comparing pathway 1 and pathway 4, pathway 4 involves two more elementary steps (smiles rearrangement) than pathway 1. It appears that pathway 1 is preferred over pathway 4. However, the rate-determining step involved in pathway 1 (potential barrier 15.25 kcal/mol, reaction heat 19.80 kcal/mol) requires crossing a higher barrier and is more endothermic than pathway 4 (potential barrier 13.63 kcal/mol, reaction heat 18.17 kcal/mol). This comparatively low barrier of the rate determining step is propitious to the occurrence of pathway 4. Therefore, pathway 1 and pathway 4 should be competitive. Similarly, pathway 5 and pathway 8 are competitive. Thus, PCTAs are preferentially formed from the 2,4-DCTP precursor from pathway 1, pathway 4, pathway 5 and pathway 8, resulting in four dominant PCTA products (2,7-DCTA, 2,8-DCTA, 1,3,8-TCTA and 1,3,7-TCTA).

To sum up all the eight pathways in Figure 1, it can be clearly seen that the rate-determining step to the PCTA formation is intra-annular elimination of Cl or H. Comparison the rate-determining step of the two steps shows that intra-annular elimination of Cl has a lower barrier and is less endothermic than intra-annular elimination of H, *i.e*., thermodynamically preferred PCTA formation pathways occur via intra-annular elimination of Cl. In addition, the Cl atom, which is eliminated, is the substituent at the *ortho*-positon in CTPs. This implies that only CTPs with chlorine atom at the *ortho*-position can form PCTA. Similar conclusion has been previously obtained in the PCDD formation from CPs. It is also interesting to compare the formation potential of PCTA from 2,4-DCTPs with that of PCDD from 2,4-DCPs [30]. The ring closure and elimination of Cl step (the rate-determining step) in PCTA formation from 2,4-DCTPs has a much lower potential barrier than PCDD formation from 2,4-DCPs by about 13 kcal/mol (with similar reaction heat) [30]. Thus, the formation of PCTAs from 2,4-DCTPs is relatively easier compared to the formation of the analogous PCDDs from 2,4-DCPs. The substitution of sulfur atom from oxygen atom enhances the elimination of Cl, e.g., enhances the formation of PCDTs.

**Figure 2:**
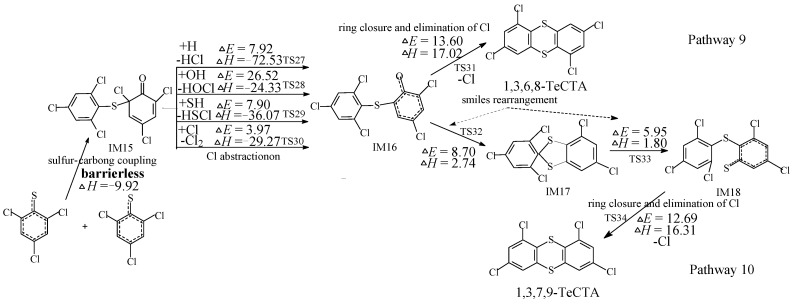
PCTA formation routes embedded with the potential barriers Δ*E* (in kcal/mol) and reaction heats Δ*H* (in kcal/mol) from dimerization of 2,4,6-TCTPRs. Δ*H* is calculated at 0 K.

Figure 2 illustrates the homogeneous gas-phase formation of PCTAs from 2,4,6-TCTPR embedded with the potential barriers and reaction heats. Due to the symmetry of 2,4,6-TCTPR, only two PCTA formation pathways (pathways 9 and 10) and two PCDT isomers (1,3,6,8-TeCTA and 1,3,7,9-TeCTA) are displayed in Figure 2 via one TPOTP intermediate IM15. 1,3,6,8-TeCDD is produced from the direct condensation of IM15 (via similar three steps as pathways 1 and 5 in Figure 1), whereas 1,3,7,9-TeCDD is formed through the condensation of IM15 after a smiles rearrangement (via similar three steps as pathways 4 and 8 in Figure 1). From Figure 2, the formation of 1,3,7,9-TeCTA involves two more elementary steps (smiles rearrangement) compared to the formation of 1,3,6,8-TeCTA. Nevertheless, the rate-determining step involved in the formation of 1,3,6,8-TeCTA (potential barrier 13.60 kcal/mol, reaction heat 17.02 kcal/mol) requires crossing a higher barrier and is more endothermic than that involved in the formation of 1,3,7,9-TeCTA (potential barrier 12.69 kcal/mol, reaction heat 16.31 kcal/mol). Thus, the formations of 1,3,6,8-TeCTA and 1,3,7,9-TeCTA should be competitive. Comparison with the previous study suggests that the potential barrier of the rate-determining rate in PCTA formation from 2,4,6-TCTP is about 17 kcal/mol lower than that in the PCDD formation from 2,4,6-TCP [29]. This reclaims the conclusion above that the substitution of sulfur atom from oxygen atom enhances the formation of PCTAs [30].

### 2.3. Formation of PCDTs from 2,4-DCTPRs

**Figure 3:**
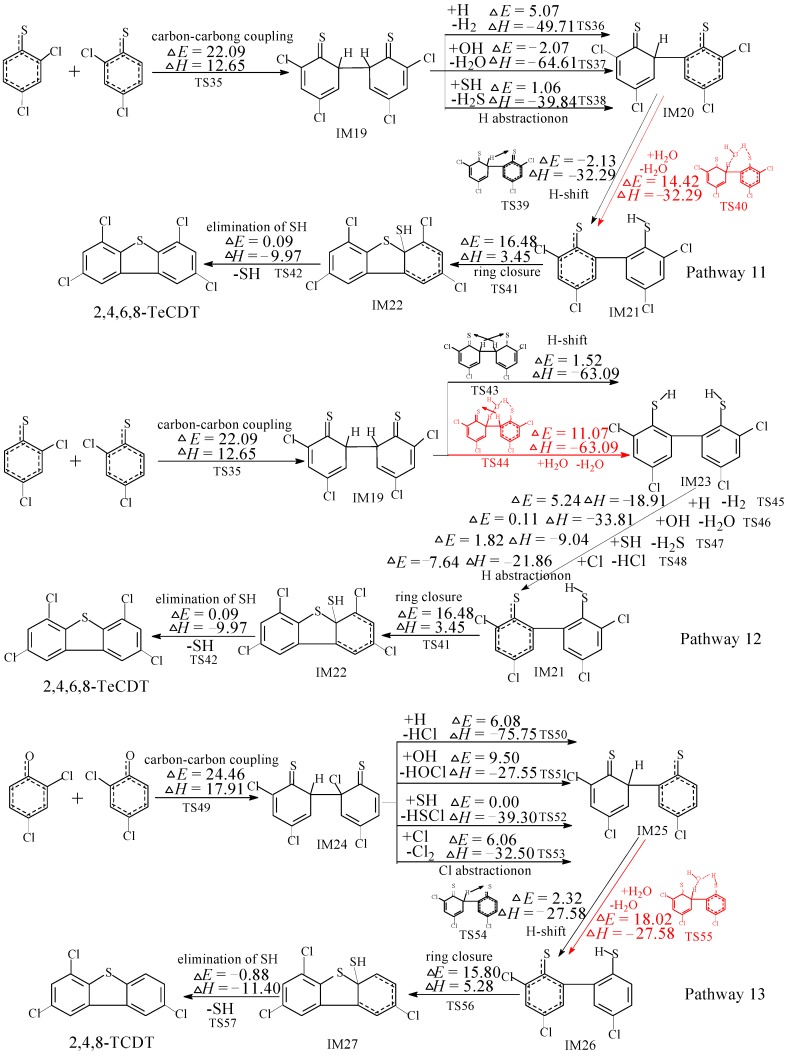
Polychlorinated thianthrene (PCTA) formation routes embedded with the potential barriers Δ*E* (in kcal/mol) and reaction heats Δ*H* (in kcal/mol) from dimerization of 2,4-DCTPRs. Δ*H* is calculated at 0 K. Reactions and energies with the water molecule participation involved in the formation of PCDTs are shown in red arrows and digits.

Figure 3 illustrates the homogeneous gas-phase formation of PCDTs from 2,4-DCTPRs embedded with embedded with the potential barriers Δ*E* (in kcal/mol) and reaction heats Δ*H* (in kcal/mol) at the MPWB1K/6-311+G(3df,2p)//MPWB1K/6-31+G(d,p) level. Two PCDT congeners (2,4,6,8-TeCDT and 2,4,8-TCDT) can be yield and three formation pathways (pathways 11–13) are proposed from dimerization of 2,4-DCTPRs. Two reaction pathways, pathways 11 and 12, are offered in Figure 3 to interpret the formation of 2,4,6,8-TeCDT. One reaction pathway, pathway 13, is proposed for the formation of 2,4,8-TCDT. In Figure 3, pathway 11 involves the following five elementary steps: (1) carbon-carbon coupling; (2) H abstraction; (3) tautomerization (H shift); (4) ring closure and (5) elimination of SH. The carbon-carbon coupling has a large potential barrier of 22.09 kcal/mol and is strongly endoergic by 12.65 kcal/mol, and it is the rate-determining step. The H abstraction step is highly exothermic. The H shift contains two steps, H shift with and without water molecular. The direct H shift step and elimination of SH step occur via a very low potential barrier and strongly exothermic. The pathway 12 also contains five elementary processes: (1) carbon-carbon coupling (the rate-determining step); (2) tautomerization (double H-shift); (3) H abstraction; (4) ring closure and (5) elimination of SH. The double H shift can occur with and without the introduction of water molecular. Pathway 13 is similar with pathway 11, involving five elementary processes: (1) carbon-carbon coupling (the rate determining step with potential barrier 24.46 kcal/mol and reaction heat 17.91 kcal/mol); (2) Cl abstraction; (3) tautomerization (H shift); (4) ring closure and (5) elimination of SH. For all the three pathways, the initial step and rate determining step are the same elementary step (carbon-carbon coupling step). In addition, the rate determining step involved in the formation of 2,4,6,8-TeCDT has a lower barrier and is less endothermic compared to those involved in the formations of 2,4,8-TCDT. Hence, pathways 11 and 12 are the thermodynamically preferred PCDT formation pathways compared to pathways 13. In addition, In addition, the formation of 2,4,6,8-TeCDT is preferred over the formations of 2,4,8-TCDT. Similar as the PCDF formation, because both *ortho*-position of 2,4,6-TCTPR are substituted with the chlorine atoms, the carbon-carbon coupling of 2,4,6-TCTPRs is sterically inhibited, e.g., no PCDT can be yield from 2,4,6-TCTP as precursor.

**Figure 4:**
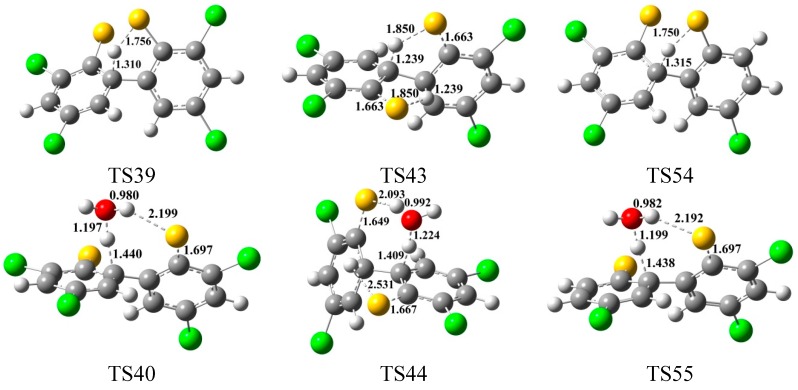
MPWB1K/6-31+G(d,p) optimized geometries for the transition states of the H shift steps with and without water molecules in the formation of PCDTs from 2,4-DCTP precursor. Distances are in angstroms. Gray sphere, C; White sphere, H; Yellow sphere, S; Red sphere, O; Green sphere, Cl. (For interpretation of the references to color in this figure legend, the reader is referred to the web version of this article.)

Previous study shows that the water molecule can obviously lower the barrier of the H-shift step involved in the formation of PCDFs [40]. The water molecule plays a positive catalytic effect on the H-shift step and the homogeneous gas-phase formation of PCDFs from CPs [40]. Naturally, it is interesting to study the role of water molecular in the formation of PCDTs from CTPs as precursor. As show in Figure 3, the water molecule can participate actively the H shift step and double H-shift step involved in the formation of PCDTs (red arrows and digits in Figure 3) through its ability to form hydrogen bonds. As seen in Figure 4, the direct H shift via the intramolecular isomerization proceeds through a five-membered ring transition state, whereas the H shift via the bimolecular reaction with the help of water proceeds through a seven-membered ring transition state. In the seven-membered transition state, the water molecule acts as a bridge, accepting the hydrogen from an aromatic ring and simultaneously donating another hydrogen atom to the oxygen keto atom of the other aromatic ring. Without water molecular, the H shift steps (IM20 → IM21, IM19 → IM23, and IM25 → IM26) are unimolecular reactions via the intramolecular isomerization. The potential barriers for the three direct H-shift steps are −2.13, 1.52, and 2.32 kcal/mol. In particular, the reactions of IM20 → IM21 exhibit negative barriers, which is due to the ZPE (zero-point energy) correction and existence of prereactive complexes. With the introduction of water, the H-shift step becomes a bimolecular reaction (IM20 + H_2_O → IM21 + H_2_O, IM19 + H_2_O → IM23 + H_2_O, and IM25 + H_2_O → IM26 + H_2_O). In addition, the potential barriers for the three bimolecular reactions become 14.42, 11.07, 18.02 kcal/mol. The barriers of the H-shift step via the bimolecular reaction with aid of water molecule are about 10–16 kcal/mol higher than direct H-shift via the intramolecular isomerization. Hence, the water molecule has a negative catalytic role on the H-shift step and hinders the formation of PCDTs. Water molecules have completely opposite effects in the formation of PCDTs and PCDFs [40].

Besides the distinct effect of water on the formation of PCDTs and PCDFs, there exist other two difference on PCDT formation mechanism from 2,4-DCTP and PCDF formation from 2,4-DCP in our previous study [30]. Firstly, the rate-determining step of PCDT formation from 2,4-DCTP is the carbon-carbon coupling step, whereas the rate-determining step of PCDF formation from 2,4-DCP is ring closure step. In addition, the potential barrier of the rate determining step of PCDT formation from 2,4-DCTP (22.09 kcal/mol in pathways 11 and 12, and 24.46 kcal/mol in pathway 13) is about 6 kcal/mol lower than that of the corresponding rate determining step of the PCDF formation from 2,4-DCP (28.41 and 31.71 kcal/mol) [30]. Second, the H-shift step and elimination of SH step in PCDT formation from 2,4-DCTP are strongly exothermic via small potential barrier. However, the two steps in PCDF formation from 2,4-DCP occur via largely higher potential barriers (about 15–20 kcal/mol higher) and less exothermic than those in the PCDT formation from 2,4-DCTP [30]. Thus, the formation of PCDTs from 2,4-DCTPs can occur readily than the formation of the similar PCDFs from 2,4-DCPs, e.g., the substitution of sulfur atom from oxygen atom promotes the formation of PCDTs [30].

### 2.4. Rate Constant Calculations

In this paper, the rate constants of the crucial elementary reactions for the formation of PCTA/DTs from 2,4-DCTP and 2,4,6-TCTP precursors were calculated by using canonical variational transition state theory (CVT) with small-curvature tunneling (SCT) contribution methods [41,42,43,44]. The CVT/SCT method is among the most promising current avenues of approach in theoretical chemical kinetics. The error correction of the kinetic calculation may be mainly from the SCT method [41,42,43,44]. Due to the absence of the available experimental rate constants, it is difficult to make a direct comparison of the calculated CVT/SCT rate constants with the experimental values for the PCTA/DTs from CTPs. Our previous research has shown that the CVT/SCT rate constants of C_6_H_5_OH + H → C_6_H_5_O + H_2_ and C_6_H_5_OH + OH → C_6_H_5_OH + H_2_O are in good agreement with the corresponding experimental values [45,46], respectively, which successfully clarified the accuracy of CVT/SCT method to calculate the rate constants.

For the formation of PCTAs, pathways ending with the elimination of Cl prefer over pathways ended with the elimination of H. This conclusion could also be reclaimed by comparing the CVT/SCT rate constants of this two steps. At a given temperature, the calculated CVT/SCT rate constants for the elementary step of elimination of Cl is larger than that of elimination of H. For example, at 1000 K, the calculated CVT/SCT rate constants is 5.49 × 10^8^ s^−1^ for reaction IM3 → 2,7-DCTA + Cl via TS5, whereas the value is 5.99 × 10^5^ s^−1^ for the reaction IM4 → 1,3,8-TCTA + H via TS7. For the formation of PCDTs, pathways for the formation of 2,4,6,8-TeCDF dominant over pathways for the formation of 2,4,8-TCDF. This could also be confirmed by comparing the CVT/SCT rate constants of rate-determining steps from 2,4,6,8-TeCDF and 2,4,8-TCDF formation pathways. For example, the CVT/SCT rate constants for reaction of 2,4-DCTP + 2,4-DCTP → IM19 via TS35 in 2,4,6,8-TeCDF formation pathways is 5.59 × 10^−21^ cm^3^·molecule·s^−1^, which is larger than CVT/SCT rate constants for the reaction of 2,4-DCTP + 2,4-DCTP → IM24 via TS49 in 2,4,8-TCDF formation pathways (2.89 × 10^−21^ cm^3^·molecule·s^−1^).

To be used more effectively, the CVT/SCT rate constants every 50 K from 600 to 1200 K were calculated for elementary reactions involved in the thermodynamically preferred formation pathways of PCTA/DTs from 2,4-DCTP and 2,4,6-TCTP precursors. The 600–1200 K covers the possible formation temperature range of PCTA/DTs in municipal waste incinerators. The calculated CVT/SCT rate constants under different temperatures are fitted in the Arrhenius form, as shown in Table 2 for the crucial elementary steps of PCTAs formation from 2,4-DCTP and 2,4,6-TCTP precursors and Table 3 for the elementary steps of PCDTs formation from 2,4-DCTP precursor. The pre-exponential factors, the activation energies, and the rate constants can be obtained from these Arrhenius.

**Table 2:** Arrhenius formulas for crucial elementary reactions involved in the formation of PCTAs from the 2,4-DCTP and 2,4,6-TCTP precursors over the temperature range of 600–1200 K (units are s^−1^ and cm^3^ molecule^−1^ s^−1^ for unimolecular and bimolecular reactions, respectively).

Reactions Arrhenius Formulas	Reactions Arrhenius Formulas
2,4-DCTP + H → 2,4-DCTPR + H_2_	2,4-DCTP + H → 2,4-DCTPR + H_2_
2,4-DCTP + OH → 2,4-DCTPR + H_2_O	2,4-DCTP + OH → 2,4-DCTPR + H_2_O
2,4-DCTP + O(3P) → 2,4-DCTPR + OH	2,4-DCTP + O(3P) → 2,4-DCTPR + OH
2,4,6-TCTP + H → 2,4,6-TCTPR + H_2_	2,4,6-TCTP + H → 2,4,6-TCTPR + H_2_
2,4,6-TCTP + OH → 2,4,6-TCTPR + H_2_O	2,4,6-TCTP + OH → 2,4,6-TCTPR + H_2_O
2,4,6-TCTP + O(3P) → 2,4,6-TCTPR + OH	2,4,6-TCTP + O(3P) → 2,4,6-TCTPR + OH
IM1 + H → IM3 + HCl via TS1	IM1 + H → IM3 + HCl via TS1
IM1 + OH → IM3 + HOCl via TS2	IM1 + OH → IM3 + HOCl via TS2
IM1 + SH → IM3 + HSCl via TS3	IM1 + SH→ IM3 + HSCl via TS3
IM1 + Cl → IM3 + Cl_2_ via TS4	IM1 + Cl → IM3 + Cl_2_ via TS4
IM3 → 2,7-DCTA + Cl via TS5	IM3 → 2,7-DCTA + Cl via TS5
IM3 → IM4 TS6	IM3 → IM4 TS6
IM4 → 1,3,8-TCTA + H via TS7	IM4 → 1,3,8-TCTA + H via TS7
IM2 + H → IM9 + H_2_ via TS14	IM2 + H → IM9 + H_2_ via TS14
IM2 + SH → IM9 + H_2_S via TS16	IM2 + SH → IM9 + H_2_S via TS16
IM9 → 1,3,8-TCTA + Cl via TS18	IM9 → 1,3,8-TCTA + Cl via TS18
IM9 → IM10 via TS19	IM9 → IM10 via TS19
IM10 → 1,3,6,8-TeCTA + H via TS20	IM10 → 1,3,6,8-TeCTA + H via TS20
IM15 + H → IM16 + HCl via TS27	IM15 + H → IM16 + HCl via TS27
IM15 + SH → IM16 + HSCl via TS29	IM15 + SH → IM16 + HSCl via TS29
IM15 + Cl → IM16 + Cl_2_ via TS30	IM15 + Cl → IM16 + Cl_2_ via TS30
IM16 → 1,3,6,8-TeCTA + Cl via TS31	IM16 → 1,3,6,8-TeCTA + Cl via TS31

**Table 3:** Arrhenius formulas for crucial elementary reactions involved in the formation of PCDTs from the 2,4-DCTP precursors over the temperature range of 600−1200 K (units are s^−1^ and cm^3^ molecule^−1^·s^−1^ for unimolecular and bimolecular reactions, respectively).

Reactions Arrhenius Formulas	Arrhenius Formulas
2,4-DCTPR + 2,4-DCTPR → IM19 via TS35	*k*(T) = (5.30 × 10^−16^) exp (−11424/T)
IM19 + H → IM20 + H_2_ via TS36	*k*(T) = (3.44 × 10^−12^) exp (−2649/T)
IM19 + SH → IM20 + H_2_S via TS38	*k*(T) = (3.49 × 10^−13^) exp (−2532/T)
IM21 → IM22 via TS41	*k*(T) = (3.07 × 10^11^) exp (−8241/T)
IM22 → 2,4,6,8-TeCDT + SH via TS42	*k*(T) = (5.86 × 10^12^) exp (−351/T)
IM19 → IM23 TS43	*k*(T) = (1.71 × 10^13^) exp (−3733/T)
IM23 + H→ IM21 + H_2_ via TS45	*k*(T) = (5.71 × 10^−9^) exp (−1148/T)
IM23 + OH → IM21 + H_2_O via TS46	*k*(T) = (7.66 × 10^−12^) exp (−1889/T)
IM23 + SH → IM21 + H_2_S via TS47	*k*(T) = (1.10 × 10^−12^) exp (−2808/T)
2,4-DCTPR + 2,4-DCTPR → IM24 via TS49	*k*(T) = (4.06 × 10^−16^) exp (−12030/T)
IM24 + H → IM25 + HCl via TS50	*k*(T) = (1.24 × 10^−11^) exp (−2972/T)
IM24 + OH → IM25 + HOCl via TS51	*k*(T) = (4.41 × 10^−14^) exp (−6968/T)
IM24 + Cl → IM25 + Cl_2_ via TS53	*k*(T) = (1.65 × 10^−12^) exp (−2463/T)
IM25 → IM26 via TS54	*k*(T) = (6.42 × 10^11^) exp (−3081/T)
IM26 → IM27 via TS56	*k*(T) = (2.25 × 10^12^) exp (−8213/T)

## 3. Experimental Section 

### 3.1. Density Functional Theory

By means of Gaussian 09 program package (Wallingford, CT, USA), high-accuracy molecular orbital calculations were carried out for all the calculations on the geometries, energies, frequencies for reactants, complexes, transition states, and products [47]. The hybrid meta function MPWB1K was employed for the homogeneous gas-phase formation of PCTA/DTs from 2,4-DCTP and 2,4,6-TCTP as precursors, which has uniformly good performance in quantum calculations of thermochemistry, thermochemical kinetics, hydrogen bonding and weak interactions [48]. The MPWB1K method has been successfully used in our previous study on the homogeneous gas-phase formation of PCDDs from CPs as precursors [28,29,30,40]. As a continuous work, this study needs to use the same method and calculation level in order to make effective comparison. The geometries of the reactants, intermediates, transition states and products were optimized by using density functional theory (DFT) at the MPWB1K/6-31+G(d,p) level [48]. The vibrational frequencies were also calculated at the same level in order to determine the nature of the stationary points, the zero-point energy (ZPE), and the thermal contributions to the free energy of activation. Besides, the intrinsic reaction coordinate (IRC) analysis was performed to confirm that each transition state connects to the right minima along the reaction path [49]. At the MPWB1K/6-31+G(d,p) level, the minimum energy paths (MEPs) were obtained in mass-weighted Cartesian coordinates. The force constant matrices of the stationary points and selected nonstationary points near the transition state along the MEP were also calculated in order to do the following kinetics calculations. Based on the optimized geometries, a more flexible basis set, 6-311+G(3df,2p), was employed to calculate the single point energies of various species. All the relative energies quoted and discussed in this work include zero-point energy (ZPE) correction with unscaled frequencies obtained at the MPWB1K/6-31+G(d,p) level.

### 3.2. Kinetic Calculation

The canonical variational transition state theory (CVT) with small-curvature tunneling (SCT) correction is an effective method to calculate the rate constants [41,42,43,44]. In this paper, the CVT/SCT method is used to calculate the rate constants of key elementary step involved in this study over a wide temperature range (600–1200 K). The CVT rate constant, *k*^CVT^(T), for temperature T is given by

(1) $$k^{\mathrm{CVT}}(T)=\underset{s}{\min}\;k^{\mathrm{GT}}(T,s)$$

where

(2) $$k^{GT}(T,s)=\frac{\text{σ}k_{\text{B}}T}{h}\frac{Q^{GT}(T,s)}{\Phi^{R}(T)}e^{-V_{MEP}(s)/k_{\text{B}}T}$$

where, *k^GT^*(*T*, *s*) is the generalized transition state theory rate constant at the dividing surface s, σ is the symmetry factor accounting for the possibility of more than one symmetry-related reaction path, *k*_B_ is Boltzmann’s constant, *h* is Planck’s constant. Ф*^R^*(*T*) is the reactant partition function per unit volume, excluding symmetry numbers for rotation, and *Q^GT^*(*T*, *s*) is the partition function of a generalized transition state at s with a local zero of energy at *V_MEP_*(*s*) and with all rotational symmetry numbers set to unity. To calculate the rate constants, 40 non-stationary points near the transition state along the minimum energy path (20 points on the reactants side and 20 points on the product side) were selected for frequency calculations at the MPWB1K/6-31+G(d,p) level. The parameters such as energy data, matrices of force constants, hassien matrixes, coordinates of each stationary points and unstationary points are obtained from the Gaussian 09 program output files and are input into the polyrate input files automatically by our self-compile program. Rate constant calculations were carried out using the Polyrate 9.7 program (University of Minnesota, Minneapolis, MN, USA) [50].

### 3.3. Accuracy Verification

It is important to verify the accuracy and reliability of the theoretical calculations, especially for a continuous work. The optimized geometries and the calculated vibrational frequencies of thiophenol, 4-chlorotriophenol and dibenzothiophene at the MPWB1K/6-31+G(d,p) level show good consistency with the available experimental values, and the relative deviation remains within 1.0% for the geometry parameters and 9.0% for the vibrational frequencies [51,52,53,54]. To verify the reliability of the energy parameters, we calculated the reaction enthalpies for the reactions of thiophenol (C_6_H_6_S) + thiophenol (C_6_H_6_S) → dibenzothiophene (C_12_H_8_S) + H_2_S + H_2_ at the MPWB1K/6-311+G(3df,2p)//MPWB1K/6-31+G(d,p) level. The calculated value of −7.60 at 298.15 K and 1.0 atm is consistent with the corresponding experimental value of −7.74 kcal/mol, obtained from the measured standard enthalpies of formation(∆*H*_f,0_) of thiophenol (26.85 kcal/mol), dibenzothiophene (50.88 kcal/mol), H_2_S (−4.92 kcal/mol), and H_2_ (0 kcal/mol) [55], especially if the experimental uncertainties are taken into consideration. From these results, the MPWB1K/6-311+G(3df,2p)//MPWB1K/6-31+G(d,p) level used in this study can extraordinarily fulfill the calculation accuracy for the species involved in the formation of PCTA/DTs from the 2,4-DCTP and 2,4,6-TCTP precursors.

## 4. Conclusions

In this study, the mechanisms of the homogeneous gas-phase formation of PCTA/DTs from 2,4-DCTP and 2,4,6-TCTP precursors were investigated theoretically using DFT electronic structure theory at the MPWB1K/6-311+G(3df,2p)//MPWB1K/6-31+G(d,p) level. Several energetically preferred routes for PCTA/DT formation were proposed. The formation potential of PCTA/DT products and role of water molecular on the mechanisms were discussed. The mechanisms were compared with the previous studies of PCDD/F formation from the 2,4-DCP and 2,4,6-TCP precursors to clarify the effect of substitution of sulfur atom from oxygen atom on the formation potential of dioxin-like compound. The kinetic calculation was performed and the rate constants were calculated over the temperature range of 600–1200 K using canonical variational transition-state (CVT) theory with the small curvature tunneling (SCT) contribution, which can support important input parameters for the PCTA/DT controlling and prediction models. Four specific conclusions can be drawn:

(1) The main PCTA products from 2,4-DCTP as precursor are 2,7-DCTA, 2,8-DCTA, 1,3,8-TCTA and 1,3,7-TCTA; The main PCTA products from 2,4,6-TCTP as precursor are 1,3,6,8-TeCTA and 1,3,7,9-TeCTA; The main PCDT product from 2,4-DCTP as precursor is 2,4,6,8-TeCDT.

(2) In the PCTA formation routes, pathways ending with elimination of the Cl step are energetically preferred to pathways ending with elimination of the H step. Only CTPs with chlorine atom at the *ortho*-position can form PCTAs.

(3) Different from the positive catalytic effect of water molecular on the formation of PCDFs from CPs, the water molecule has a negative catalytic effect on the H-shift step and hinders the formation of PCDTs from 2,4-DCTP.

(4) The formation of PCTA/DTs from 2,4-DCTPs and 2,4,6-TCTPs is easier compared to the formation of the analogous PCDD/Fs from 2,4-DCPs and 2,4,6-TCPs.

## Supplementary Materials

Electron density from total SCF density of 2,4-DCTPR, 2,4-DCPR, 2,4,6-TCTPR 2,4,6-TCPR, at MPWB1K/6-31+G(d,p) level. The imaginary frequencies, the zero-point energies and the total energies for the transition states involved in the formation of PCTA/DTs from the 2,4-DCTP and 2,4,6-TCTP as precursors. Cartesian coordinates for the reactants, intermediates, transition states and products involved in formation of PCTA/DTs from the 2,4-DCTP and 2,4,6-TCTP as precursors. Supplementary materials can be found at http://www.mdpi.com/1422-0067/16/09/20449/s1.
