# Supplementary material for: Mechanistic and Kinetic Studies on the Homogeneous Gas-Phase Formation of PCTA/DTs from 2,4-Dichlorothiophenol and 2,4,6-Trichlorothiophenol
Source: Int J Mol Sci. 2015 Aug 28;16(9):20449–67. doi: 10.3390/ijms160920449 (PMC4613213; doi:10.3390/ijms160920449)
Supplement: Supplementary file 1 [file ijms-16-20449-s001.pdf]

Supplementary Information

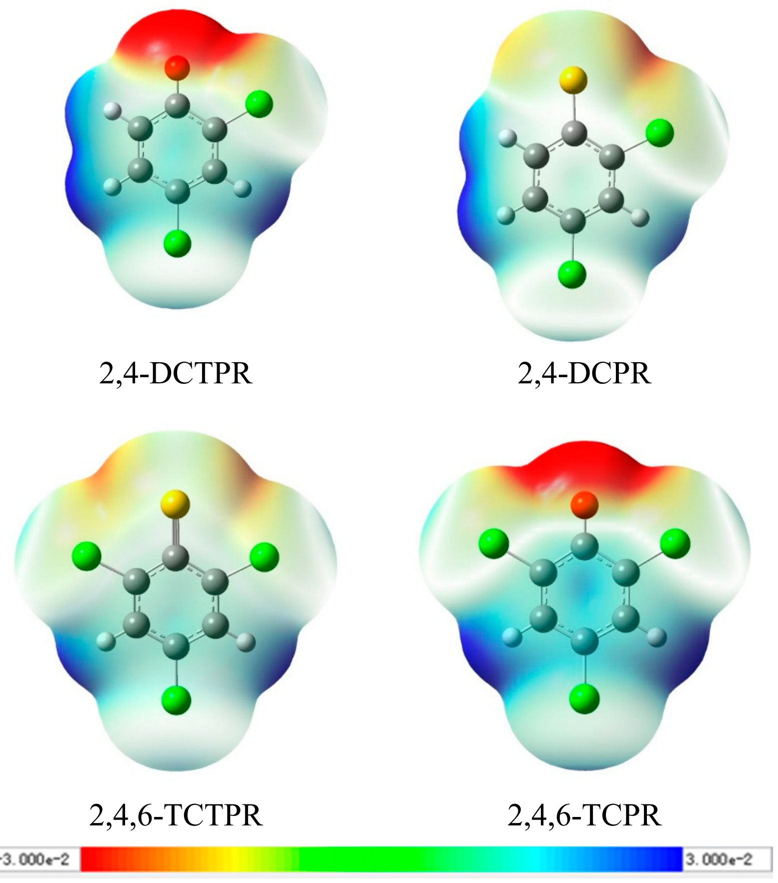

Figure S1. Electron density from total SCF density of 2,4-DCTPR, 2,4-DCPR, 2,4,6-TCTPR 2,4,6-TCPR, at MPWB1K/6-31+G(d,p) level. This is mapped on the surface of molecular electron density at 0.003 e a.u.$^{-3}$.

Table S1. Imaginary frequencies (in cm^{-1}), zero point energies (ZPE, in a.u.) and total energies (in a.u.) for the transition states involved in the formation of PCTA/DTs from the 2,4-DCTP and 2,4,6-TCTP as precursors.

Transition States	Imaginary Frequencies	ZPE	Total Energies
TS1	999i	0.15014	−3098.87118
TS2	486i	0.15917	−3174.09054
TS3	440i	0.15611	−3497.17147
TS4	338i	0.14810	−3558.58728
TS5	423i	0.14671	−2638.09754
TS6	351i	0.14684	−2638.10371
TS7	1071i	0.13978	−2638.05447
TS8	411i	0.14659	−2638.10371
TS9	407i	0.14659	−2638.10384
TS10	344i	0.14683	−2638.10739
TS11	1071i	0.13979	−2638.05440
TS12	406i	0.14666	−2638.10455
TS13	414i	0.14663	−2638.09743
TS14	1068i	0.14811	−3098.87334

Table S1. *Cont.*

Transition States	Imaginary Frequencies	ZPE	Total Energies
TS15	661i	0.15726	−3174.12023
TS16	590i	0.15274	−3497.18203
TS17	557i	0.14717	−3558.59951
TS18	418i	0.13672	−3097.77654
TS19	343i	0.13700	−3097.78604
TS20	1069i	0.12983	−3097.73282
TS21	398i	0.13685	−3097.78500
TS22	398i	0.13664	−3097.78384
TS23	331i	0.13698	−3097.78581
TS24	1074i	0.12986	−3097.73233
TS25	392i	0.13676	−3097.78493
TS26	411i	0.13676	−3097.77690
TS27	995i	0.13039	−4018.22290
TS28	579i	0.13919	−4093.42125
TS29	478i	0.13631	−4416.52501
TS30	405i	0.12825	−4477.93778
TS31	420i	0.12678	−3557.45433
TS32	397i	0.12695	−3557.46230
TS33	396i	0.12685	−3557.46226
TS34	411i	0.12685	−3557.45436
TS35	225i	0.14831	−3098.32894
TS36	986i	0.14794	−3098.83553
TS37	431i	0.15743	−3174.07559
TS38	930i	0.15253	−3497.14266
TS39	1279i	0.13363	−3097.75586
TS40	1276i	0.15812	−3174.15298
TS41	361i	0.13465	−3097.77868
TS42	424i	0.13439	−3097.79903
TS43	910i	0.14176	−3098.33501
TS44	1251i	0.17006	−3174.74709
TS45	927i	0.14488	−3098.93274
TS46	865i	0.15488	−3174.17012
TS47	1034i	0.14931	−3497.23877
TS48	877i	0.14322	−3558.66233
TS49	355i	0.14894	−3098.32579
TS50	874i	0.15043	−3098.82804
TS51	599i	0.16010	−3174.05147
TS52	392i	0.14860	−3497.13206
TS53	493i	0.15684	−3558.54519
TS54	1280i	0.14365	−2638.07649
TS55	1342i	0.16832	−2714.47514
TS56	367i	0.14420	−2638.09954
TS41	361i	0.13465	−3097.77868
TS57	424i	0.14420	−2638.11768

Table S2. Cartesian coordinates for the reactants, intermediates and products involved in the formation of PCTA/DTs from the 2,4-DCTP and 2,4,6-TCTP as precursors.

IM	*x*	*y*	*z*
IM1			
0	2	-	-
C	1.367229	0.661133	−0.175931
C	2.493692	0.864789	0.624245
C	3.628436	0.088132	0.502513
C	3.647708	−0.924058	−0.435080
C	2.566943	−1.151050	−1.261685
C	1.451247	−0.344479	−1.140303
H	4.477922	0.268117	1.138683
C	−1.390763	−0.376394	1.142011
C	−2.515855	−1.213402	1.230398
C	−3.625673	−0.971809	0.458235
C	−3.642775	0.082684	−0.437875
C	−2.519487	0.881245	−0.573795
C	−1.370856	0.666856	0.180514
H	−2.501349	−2.025406	1.937850
H	2.593567	−1.928077	−2.006337
Cl	0.161181	−0.616607	−2.243878
H	−4.512375	0.273748	−1.044026
S	−0.139454	−0.613674	2.275785
S	−0.007303	1.753091	−0.038377
Cl	5.048701	−1.911162	−0.585861
Cl	−5.012804	−1.980555	0.598821
Cl	2.508402	2.099755	1.816581
Cl	−2.602464	2.149690	−1.732968
IM2			
0	1	-	-
C	1.505910	−0.778907	0.516850
C	2.181246	0.414524	0.773096
C	3.507206	0.574749	0.408337
C	4.165430	−0.464028	−0.220068
C	3.526170	−1.661917	−0.484734
C	2.207089	−1.805725	−0.108073
Cl	1.401664	1.738976	1.544366
H	4.015849	1.501532	0.613809
H	1.694990	−2.737930	−0.290879
C	−1.017493	−0.697877	−0.620838
C	−0.779615	0.722411	−0.944419
C	−1.728412	1.627039	−0.707049
C	−3.013614	1.245983	−0.192744
C	−3.348162	−0.049243	−0.026814
C	−2.443002	−1.116440	−0.392033
H	−3.726419	2.019244	0.043600

Table S2. *Cont.*

IM	*x*	*y*	*z*
IM2			
H	−0.570190	−1.365429	−1.350042
Cl	−4.918346	−0.441973	0.534523
H	4.055981	−2.466062	−0.967889
H	0.195775	1.011067	−1.302657
S	−0.166659	−1.064921	0.995622
S	−2.864692	−2.679104	−0.507182
Cl	−1.457674	3.307360	−0.968008
Cl	5.813128	−0.258565	−0.673824
IM3			
0	2	-	-
C	−1.068838	0.471030	−0.362514
C	−2.345703	1.018443	−0.303858
C	−3.474293	0.220170	−0.314169
C	−3.328822	−1.149518	−0.385003
C	−2.072781	−1.722644	−0.458861
C	−0.957292	−0.910356	−0.456128
Cl	−2.557919	2.726678	−0.226516
H	−4.451698	0.669671	−0.262980
H	0.019452	−1.362215	−0.526931
C	1.642288	0.503307	0.095261
C	2.676397	0.356437	−0.808138
C	3.802226	−0.380414	−0.465136
C	3.903482	−1.002360	0.769523
C	2.869140	−0.866890	1.665805
C	1.724917	−0.100351	1.378124
H	2.606968	0.808593	−1.783997
H	2.925821	−1.341074	2.632607
H	−1.968582	−2.793133	−0.524768
H	4.779339	−1.580520	1.014283
S	0.316040	1.560482	−0.381500
S	0.526883	0.087474	2.571378
Cl	−4.732439	−2.151743	−0.388400
Cl	5.077276	−0.539038	−1.603653
IM4			
0	2	-	-
C	−1.037923	0.650219	−0.094248
C	−2.241108	1.287344	0.057676
C	−3.457086	0.620738	−0.071262
C	−3.445931	−0.753503	−0.382923
C	−2.301446	−1.448770	−0.548201

Table S2. *Cont.*

IM	*x*	*y*	*z*
IM4			
C	−0.985377	−0.804240	−0.364163
Cl	−2.275996	2.973364	0.420041
H	−4.384995	1.149202	0.058135
H	−0.340429	−1.005103	−1.223158
C	1.683790	0.271374	0.091482
C	2.943109	0.697837	−0.318290
C	4.022900	−0.151472	−0.210002
C	3.871256	−1.438914	0.275656
C	2.612542	−1.872039	0.638506
C	1.510306	−1.030297	0.564262
H	3.078012	1.694115	−0.708641
H	2.473554	−2.885058	0.983102
H	4.722016	−2.096368	0.344672
H	−2.318785	−2.495297	−0.803666
S	0.446657	1.519345	0.105492
S	−0.070618	−1.620947	1.033723
Cl	−4.970111	−1.540066	−0.579943
Cl	5.581624	0.395789	−0.705324
IM5			
0	2	-	-
C	−1.038276	−0.545053	−0.138449
C	−1.621348	0.737633	0.334822
C	−2.952001	1.013435	0.301826
C	−3.854530	0.065707	−0.189230
C	−3.399588	−1.183319	−0.649652
C	−2.082089	−1.480478	−0.627024
Cl	−0.539364	1.917920	0.942626
H	−3.309958	1.966080	0.656171
H	−1.724952	−2.434970	−0.978346
C	1.613420	−0.286400	−0.542298
C	2.829340	0.185630	−0.999258
C	3.929650	0.102025	−0.164692
C	3.828688	−0.420066	1.110830
C	2.601172	−0.870594	1.566259
C	1.495447	−0.814825	0.738844
H	2.926110	0.614538	−1.983219
H	2.512321	−1.262702	2.567160
H	−4.111365	−1.902865	−1.020758
S	−0.085963	−1.405643	1.193490
S	0.165789	−0.281433	−1.519766
H	4.697537	−0.463433	1.746434
Cl	5.455813	0.680171	−0.729685

Table S2. *Cont.*

IM	*x*	*y*	*z*
IM5			
Cl	−5.526228	0.428638	−0.225817
IM6			
0	2	-	-
C	1.231547	0.542295	−0.410161
C	2.532030	0.914246	−0.087485
C	3.549944	−0.017907	−0.006352
C	3.267798	−1.345491	−0.253400
C	1.987870	−1.743549	−0.591707
C	0.985883	−0.798989	−0.675883
H	4.547469	0.295986	0.251357
C	−1.834330	0.156308	0.815202
C	−3.069228	−0.511577	0.862712
C	−3.950603	−0.436563	−0.192153
C	−3.626921	0.280047	−1.332917
C	−2.395470	0.912095	−1.412946
C	−1.493537	0.863799	−0.365648
H	−3.321878	−1.070803	1.748490
H	1.778882	−2.781079	−0.793957
H	−2.128953	1.446598	−2.310974
Cl	2.912066	2.567217	0.215969
H	−0.008250	−1.113998	−0.951144
S	−0.820602	0.102679	2.186482
S	−0.006583	1.791792	−0.526370
Cl	4.531035	−2.514344	−0.144042
H	−4.321903	0.327679	−2.155529
Cl	−5.467824	−1.247237	−0.098139
IM7			
0	2	-	-
C	1.187883	0.707504	−0.161657
C	2.486865	1.085363	0.058735
C	3.539993	0.175645	0.031431
C	3.258755	−1.177086	−0.247865
C	2.006668	−1.623929	−0.476372
C	0.847263	−0.712591	−0.399123
Cl	2.850869	2.740194	0.380825
H	4.548641	0.502755	0.212336
H	0.228315	−0.806956	−1.294708
C	−1.693758	−0.384519	0.361333
C	−2.945142	−0.986214	0.388166
C	−4.050201	−0.300457	−0.071365
C	−3.927984	0.971457	−0.604188
C	−2.681626	1.557832	−0.655628
C	−1.559039	0.904811	−0.156558

Table S2. *Cont.*

IM	*x*	*y*	*z*
IM7			
H	−3.047870	−1.991834	0.762789
H	−2.575214	2.545705	−1.077538
H	1.818968	−2.659990	−0.705051
S	−0.303819	−1.271786	0.951661
S	−0.092395	1.870329	−0.085779
Cl	4.592393	−2.270989	−0.319473
H	−4.795130	1.487707	−0.981261
Cl	−5.599517	−1.055145	−0.012653
**IM8**			
0	2	-	-
C	−1.374678	−0.018124	1.016938
C	−2.238487	1.068421	1.087875
C	−3.375331	1.141660	0.309167
C	−3.644881	0.114282	−0.576088
C	−2.810832	−0.981449	−0.672569
C	−1.690339	−1.048866	0.136086
H	−4.035497	1.990521	0.374737
C	1.658431	1.081187	0.176157
C	2.792925	1.025859	−0.653584
C	3.657972	−0.040459	−0.587544
C	3.414712	−1.095193	0.279511
C	2.275354	−1.079268	1.065996
C	1.387882	−0.018954	1.025434
H	2.984276	1.847531	−1.323761
H	−3.032441	−1.782005	−1.357901
Cl	−0.712379	−2.460666	0.017081
H	−1.989002	1.875663	1.757807
H	2.059367	−1.917656	1.709272
H	4.096521	−1.929235	0.318293
S	0.716124	2.502053	0.159217
S	0.003285	−0.077903	2.121184
Cl	−5.045363	0.193960	−1.577016
Cl	5.057147	−0.072459	−1.593543
IM9			
0	2	-	-
C	1.423150	−0.792115	0.078271
C	2.696103	−0.938621	−0.460372
C	3.767097	−0.205326	0.017004
C	3.565078	0.690014	1.046736
C	2.312310	0.847964	1.610718
C	1.257822	0.099669	1.130733
Cl	2.980229	−2.048788	−1.745904
H	4.743405	−0.332135	−0.419764

Table S2. *Cont.*

IM	*x*	*y*	*z*
IM9			
H	0.283726	0.214841	1.580236
C	−1.307810	−0.790199	−0.200959
C	−2.282649	−1.370664	0.582121
C	−3.470211	−0.692620	0.817537
C	−3.685836	0.573780	0.308657
C	−2.705702	1.159824	−0.464548
C	−1.491403	0.500353	−0.778422
H	−4.603652	1.098443	0.512705
Cl	−3.028508	2.730553	−1.065159
H	2.166381	1.541902	2.422029
H	−2.120002	−2.343115	1.016458
S	−0.338659	1.195555	−1.807425
S	0.105001	−1.784661	−0.542313
Cl	4.894490	1.614608	1.637793
Cl	−4.681447	−1.428525	1.782641
IM10			
0	2	-	-
C	1.366522	−0.837203	0.064815
C	2.621366	−1.315815	−0.204613
C	3.764408	−0.544633	−0.013367
C	3.621280	0.766061	0.485907
C	2.417726	1.305220	0.770886
C	1.170294	0.550814	0.532336
Cl	2.813149	−2.922835	−0.798651
H	4.737340	−0.945982	−0.235866
H	0.541229	0.565370	1.425667
C	−1.385644	−0.726133	−0.005443
C	−2.570281	−1.348344	0.370403
C	−3.745413	−0.629512	0.375628
C	−3.764108	0.708056	0.031017
C	−2.575277	1.324200	−0.303874
C	−1.363682	0.635039	−0.327287
H	−4.683315	1.268536	0.036291
Cl	−2.621884	2.994549	−0.717411
H	2.335178	2.303643	1.167499
H	−2.573097	−2.391897	0.641246
S	0.131974	1.453203	−0.718602
S	−0.028363	−1.824456	−0.206060
Cl	−5.215097	−1.406772	0.824251
Cl	5.061582	1.677051	0.756592
IM11			
0	2	-	-
C	−1.271681	−0.061473	−0.559643

Table S2. *Cont.*

IM	*x*	*y*	*z*
IM11			
C	−1.864081	−0.255573	0.790555
C	−3.202479	−0.296610	1.022180
C	−4.104016	−0.149404	−0.035724
C	−3.639651	0.043453	−1.349664
C	−2.314139	0.086153	−1.606388
Cl	−0.781606	−0.435514	2.104717
H	−3.567512	−0.441481	2.025637
H	−1.950121	0.233950	−2.610244
C	1.315681	0.618266	−0.362430
C	2.494384	1.251941	−0.014296
C	3.669005	0.537950	0.148096
C	3.645913	−0.829557	−0.035490
C	2.482210	−1.497063	−0.376305
C	1.326379	−0.761530	−0.547295
H	2.485820	−2.567162	−0.501234
H	−4.350883	0.157401	−2.151657
H	4.579362	1.045303	0.418019
Cl	2.509038	2.958850	0.206790
S	−0.197459	1.444967	−0.619777
S	−0.186536	−1.480625	−1.039157
Cl	5.100592	−1.731257	0.175153
Cl	−5.785548	−0.202479	0.273389
IM12			
0	2	-	-
C	1.515165	0.498311	0.789281
C	2.501800	−0.148656	1.523364
C	3.686741	−0.550964	0.942605
C	3.879389	−0.322935	−0.407759
C	2.919354	0.313957	−1.168064
C	1.749891	0.737919	−0.561568
H	4.444558	−1.050655	1.522792
C	−1.281549	−1.140225	0.703415
C	−2.327072	−1.741565	−0.014813
C	−3.339695	−0.972287	−0.536149
C	−3.325693	0.403142	−0.392556
C	−2.267642	1.010728	0.266270
C	−1.221682	0.273127	0.806911
H	−2.336505	−2.813268	−0.121304
H	3.080007	0.495845	−2.217331
H	−4.116064	1.006062	−0.807098
Cl	−2.298281	2.726411	0.368345
S	0.091036	1.095841	1.643201

Table S2. *Cont.*

IM	*x*	*y*	*z*
IM12			
S	−0.178888	−2.171385	1.500686
Cl	−4.640375	−1.717417	−1.382117
H	2.315989	−0.351413	2.566090
Cl	0.602555	1.578860	−1.527764
Cl	5.341018	−0.837334	−1.161461
IM13			
0	2	-	-
C	1.369631	0.572010	−0.055751
C	2.619985	1.054146	0.235246
C	3.763675	0.271682	0.106811
C	3.632111	−1.053315	−0.356756
C	2.435674	−1.597258	−0.659884
C	1.185882	−0.834413	−0.468997
Cl	2.808670	2.680754	0.773722
H	4.730762	0.676646	0.347569
H	0.562496	−0.891711	−1.363963
C	−1.353070	−0.854133	0.384693
C	−2.519217	−1.605575	0.405222
C	−3.722878	−1.015121	0.086718
C	−3.783011	0.312224	−0.289258
C	−2.616376	1.047164	−0.327544
C	−1.379605	0.496098	0.017237
H	−2.477065	−2.651070	0.663416
H	2.359869	−2.606669	−1.029038
H	−4.720214	0.772410	−0.551897
Cl	−2.729919	2.705341	−0.780350
S	−0.015609	1.598375	0.104041
S	0.139552	−1.657305	0.826418
Cl	−5.169649	−1.947876	0.129541
Cl	5.077031	−1.974558	−0.559376
IM14			
0	2	-	-
C	1.515168	0.498665	0.789049
C	2.501740	−0.148114	1.523386
C	3.686687	−0.550655	0.942865
C	3.879508	−0.323046	−0.407585
C	2.919530	0.313761	−1.168079
C	1.750061	0.737982	−0.561827
H	4.444501	−1.050155	1.523228
C	−1.281513	−1.140109	0.703561
C	−2.326974	−1.741587	−0.014582
C	−3.339667	−0.972494	−0.536158

Table S2. *Cont.*

IM	*x*	*y*	*z*
IM14			
C	−3.325763	0.402955	−0.392665
C	−2.267764	1.010690	0.266115
C	−1.221715	0.273287	0.806734
H	−2.336480	−2.813320	−0.120815
H	3.080331	0.495313	−2.217387
Cl	0.602981	1.578822	−1.528372
H	2.315826	−0.350508	2.566171
H	−4.116222	1.005721	−0.807269
Cl	−2.298851	2.726388	0.368383
S	0.091010	1.096294	1.642844
S	−0.178958	−2.171144	1.501120
Cl	−4.640236	−1.717862	−1.382005
Cl	5.341014	−0.837786	−1.161079
IM15			
0	1	-	-
C	−1.235864	−0.465730	−0.765507
C	−1.639550	0.865596	−0.934812
C	−2.914613	1.291808	−0.615782
C	−3.819286	0.370046	−0.132593
C	−3.488220	−0.964427	−0.004016
C	−2.211616	−1.372615	−0.334936
Cl	−0.570896	2.035834	−1.596815
H	−3.190835	2.323936	−0.745504
C	1.311761	−0.899011	0.446836
C	2.660680	−1.351554	0.027681
C	3.600450	−0.456386	−0.264085
C	3.376106	0.955455	−0.113475
C	2.238855	1.418759	0.431409
C	1.196696	0.530382	0.927795
Cl	0.675971	−2.041037	1.638602
H	2.811812	−2.410509	−0.096342
H	4.149454	1.639919	−0.420264
H	−4.216069	−1.682127	0.333235
S	0.386483	−0.980902	−1.179923
S	−0.031316	1.032846	1.853553
Cl	−5.404266	0.885972	0.285177
Cl	5.150310	−0.941084	−0.828371
Cl	−1.899345	−3.059640	−0.271084
Cl	2.066494	3.106057	0.663062
IM16			
0	2	-	-
C	1.384259	−0.499331	0.766957

Table S2. *Cont.*

IM	*x*	*y*	*z*
IM16			
C	2.492139	0.285391	1.097294
C	3.620496	0.329425	0.302162
C	3.655350	−0.425657	−0.852330
C	2.597011	−1.235433	−1.206519
C	1.486060	−1.278054	−0.385598
Cl	2.491089	1.247808	2.518646
H	4.453043	0.952756	0.580049
C	−1.367712	−0.148040	0.802276
C	−2.443953	−1.008859	0.862449
C	−3.588298	−0.727644	0.136032
C	−3.666245	0.384598	−0.684235
C	−2.591445	1.241608	−0.745808
C	−1.413488	1.040871	0.020706
H	−2.382751	−1.911866	1.447261
H	2.635866	−1.838908	−2.097101
H	−4.551455	0.576335	−1.266126
S	−0.008545	−0.571437	1.843646
S	−0.169437	2.187544	0.048189
Cl	5.049010	−0.366357	−1.859413
Cl	−4.922429	−1.802663	0.218061
Cl	0.222301	−2.351443	−0.844232
Cl	−2.739502	2.602697	−1.775049
IM17			
0	2	-	-
C	1.133010	0.008970	0.237753
C	1.705222	−0.845163	−0.843789
C	3.032259	−1.008784	−1.065993
C	3.962835	−0.354891	−0.249972
C	3.540974	0.466291	0.801567
C	2.218403	0.643358	1.045034
Cl	0.581789	−1.650494	−1.852755
H	3.368610	−1.640997	−1.870780
C	−1.458825	0.579740	−0.185870
C	−2.653152	1.020994	−0.726671
C	−3.848668	0.390281	−0.432028
C	−3.832563	−0.701624	0.412045
C	−2.655335	−1.174006	0.964708
C	−1.475534	−0.520444	0.665410
H	4.268470	0.961056	1.423166
S	0.053559	−0.996833	1.353823
S	0.074747	1.343461	−0.493863
Cl	5.634675	−0.562922	−0.539908

Table S2. *Cont.*

IM	*x*	*y*	*z*
IM17			
Cl	1.736755	1.648848	2.342011
H	−4.770051	0.746211	−0.860307
H	−2.666579	−2.034321	1.613219
Cl	−2.654879	2.385555	−1.775748
Cl	−5.313934	−1.503713	0.779470
IM18			
0	2	-	-
C	1.367229	0.661133	−0.175931
C	2.493692	0.864789	0.624245
C	3.628436	0.088132	0.502513
C	3.647708	−0.924058	−0.435080
C	2.566943	−1.151050	−1.261685
C	1.451247	−0.344479	−1.140303
H	4.477922	0.268117	1.138683
C	−1.390763	−0.376394	1.142011
C	−2.515855	−1.213402	1.230398
C	−3.625673	−0.971809	0.458235
C	−3.642775	0.082684	−0.437875
C	−2.519487	0.881245	−0.573795
C	−1.370856	0.666856	0.180514
H	−2.501349	−2.025406	1.937850
H	2.593567	−1.928077	−2.006337
Cl	0.161181	−0.616607	−2.243878
H	−4.512375	0.273748	−1.044026
S	−0.139454	−0.613674	2.275785
S	−0.007303	1.753091	−0.038377
Cl	5.048701	−1.911162	−0.585861
Cl	−5.012804	−1.980555	0.598821
Cl	2.508402	2.099755	1.816581
Cl	−2.602464	2.149690	−1.732968
IM19			
0	1	-	-
C	−1.822650	1.128180	−1.483216
C	−3.021978	0.772449	−0.776536
C	−3.047121	−0.241043	0.111570
C	−1.867765	−1.017835	0.429317
C	−0.648873	−0.738396	−0.415886
C	−0.697638	0.438890	−1.316138
H	−3.918361	1.341981	−0.962320
H	−0.543085	−1.617556	−1.068929
Cl	−4.516878	−0.583050	0.924961
H	3.918294	1.341765	0.962602
C	3.021934	0.772243	0.776682

Table S2. *Cont.*

IM	*x*	*y*	*z*
IM19			
C	1.822651	1.127611	1.483620
C	3.047073	−0.240928	−0.111790
C	0.697674	0.438299	1.316395
C	1.867743	−1.017675	−0.429728
Cl	4.516800	−0.582587	−0.925382
C	0.648879	−0.738604	0.415645
H	0.543064	−1.618041	1.068303
H	0.197677	0.688507	−1.865139
H	−0.197597	0.687617	1.865605
S	−1.847341	−2.161268	1.589914
S	1.847425	−2.160975	−1.590458
Cl	1.920675	2.471506	2.557297
Cl	−1.920650	2.472512	−2.556348
IM20			
0	2	-	-
C	0.685190	0.984744	0.622383
C	1.886388	1.485476	1.095844
C	3.094952	0.895998	0.764127
C	3.090390	−0.219930	−0.038870
C	1.886967	−0.784772	−0.538893
C	0.677169	−0.122464	−0.195433
H	4.022509	1.302420	1.129638
C	−0.653282	−0.671030	−0.667183
C	−1.596467	0.426492	−1.105683
C	−1.145621	−1.621324	0.368123
C	−2.875830	0.481777	−0.433653
C	−2.347121	−1.471890	0.917927
H	−0.490244	−2.431304	0.648045
C	−3.224060	−0.402883	0.521022
H	−4.186980	−0.323220	0.999360
H	−0.436419	−1.264452	−1.559260
H	−0.237159	1.476923	0.887001
Cl	4.605302	−0.915944	−0.426336
Cl	−3.987634	1.717019	−0.855295
S	1.892439	−2.188481	−1.487782
S	−1.147464	1.466589	−2.270784
Cl	−2.939306	−2.554672	2.121691
Cl	1.882616	2.865876	2.111351
IM21			
0	2	-	-
C	−2.760973	1.278247	−0.883366
C	−3.531278	0.392843	−0.152356

Table S2. *Cont.*

IM	*x*	*y*	*z*
IM21			
C	−2.902919	−0.543956	0.640240
C	−1.492441	−0.626080	0.728688
C	−0.743416	0.300536	−0.047474
C	−1.374205	1.237501	−0.836815
H	−4.606308	0.434257	−0.198307
H	−0.423381	−1.565770	−1.518007
Cl	−3.898024	−1.613746	1.531353
H	4.583607	0.489383	0.237401
C	3.509360	0.448770	0.174052
C	2.723843	1.389525	0.804402
C	2.895250	−0.552697	−0.551507
C	1.347625	1.325172	0.719693
C	1.511029	−0.648873	−0.660948
Cl	3.892123	−1.712952	−1.343392
C	0.738073	0.306582	0.005856
H	−0.794284	1.934013	−1.420163
H	0.741868	2.058820	1.226461
S	0.848047	−1.955041	−1.624688
S	−0.707818	−1.759203	1.722302
Cl	3.468037	2.655788	1.706503
Cl	−3.536540	2.449251	−1.866933
IM22			
0	2	-	-
C	3.136266	1.334381	0.033057
C	3.582267	0.042303	−0.200841
C	2.659529	−0.981700	−0.309105
C	1.308132	−0.720948	−0.180484
C	0.875329	0.591590	0.045176
C	1.793997	1.626972	0.153988
H	4.634882	−0.165114	−0.294730
H	−0.061383	−0.822411	2.431230
Cl	3.194260	−2.586470	−0.616600
H	−4.315976	0.226829	−0.746133
C	−3.285320	0.322923	−0.447107
C	−2.710742	1.606024	−0.321566
C	−2.531937	−0.778514	−0.231920
C	−1.344897	1.772357	−0.124499
C	−1.163775	−0.668932	0.319872
Cl	−3.181672	−2.349079	−0.423122
C	−0.559736	0.670611	0.075968
H	1.472763	2.638936	0.339694
H	−0.909919	2.755551	−0.208806

Table S2. *Cont.*

IM	*x*	*y*	*z*
IM22			
S	0.025881	−1.893328	−0.320584
S	−1.372890	−0.885574	2.178786
Cl	4.296994	2.601664	0.178616
Cl	−3.710930	2.976605	−0.558171
IM23			
0	1	-	-
C	2.732586	1.331022	0.885870
C	3.516078	0.439507	0.182836
C	2.901209	−0.516944	−0.599389
C	1.514244	−0.615214	−0.700024
C	0.740907	0.296228	0.031084
C	1.355905	1.263353	0.812432
H	4.590844	0.484544	0.235750
H	0.430606	−1.536594	1.515533
Cl	3.895885	−1.613608	−1.478436
H	−4.590834	0.484573	−0.235770
C	−3.516069	0.439529	−0.182846
C	−2.732564	1.331048	−0.885859
C	−2.901214	−0.516938	0.599370
C	−1.355885	1.263371	−0.812406
C	−1.514251	−0.615218	0.700022
Cl	−3.895906	−1.613603	1.478399
C	−0.740900	0.296234	−0.031060
H	−0.430635	−1.536627	−1.515405
H	0.749764	1.959954	1.368692
H	−0.749734	1.959974	−1.368652
S	0.845836	−1.860221	−1.735756
S	−0.845872	−1.860239	1.735758
Cl	3.481608	2.535222	1.863774
Cl	−3.481568	2.535264	−1.863757
IM24			
0	1	-	-
C	−0.892152	1.311928	−1.533083
C	−2.006741	1.179430	−0.627565
H	0.749764	1.959954	1.368692
C	−2.284215	−0.001865	−0.049178
C	−1.483985	−1.182334	−0.331862
C	−0.111799	−0.911534	−0.888752
C	−0.006490	0.333723	−1.700972
H	−2.641693	2.033191	−0.455650

Table S2. *Cont.*

IM	*x*	*y*	*z*
IM24			
H	0.210137	−1.774939	−1.470114
Cl	−3.657664	−0.141193	0.966136
H	−0.313459	0.009417	1.835788
C	0.580224	0.204751	1.265087
C	1.295449	1.309188	1.453522
C	0.975880	−0.816448	0.258039
C	2.522070	1.547412	0.735383
C	2.321735	−0.584184	−0.425858
Cl	1.079049	−2.366055	1.142850
C	3.006862	0.641755	−0.131612
H	3.941305	0.810673	−0.641643
H	0.820605	0.436834	−2.384298
H	3.056964	2.464089	0.927671
S	2.916712	−1.654163	−1.496530
S	−1.997512	−2.693922	−0.073121
Cl	0.813508	2.500918	2.598805
Cl	−0.780898	2.794517	−2.404040
IM25			
0	2	-	-
C	−2.037252	1.650840	−0.665380
C	−2.821263	0.853485	0.231397
C	−2.432591	−0.385799	0.600368
C	−1.210491	−0.974436	0.122529
C	−0.371555	−0.141897	−0.815322
C	−0.879101	1.204278	−1.149913
H	−3.745589	1.258145	0.611324
H	−0.389370	−0.745167	−1.745716
Cl	−3.425731	−1.275193	1.679638
H	1.044083	1.813137	0.471759
C	1.648295	0.957375	0.213151
C	2.992515	0.943640	0.533885
C	1.088383	−0.127682	−0.438920
C	3.786202	−0.146966	0.224283
C	1.868928	−1.247701	−0.750319
C	3.218512	−1.238467	−0.398782
H	3.820821	−2.105219	−0.619060
H	−0.285667	1.806003	−1.820376
H	4.831975	−0.144022	0.485448
S	−0.757644	−2.497447	0.503878
S	1.160318	−2.620278	−1.535857
Cl	3.684554	2.303927	1.338095
Cl	−2.660150	3.201896	−1.083239

Table S2. *Cont.*

IM	*x*	*y*	*z*
IM26			
0	2	-	-
C	2.234755	1.667835	0.315014
C	3.078956	0.707204	−0.209776
C	2.549284	−0.501889	−0.608526
C	1.168173	−0.788507	−0.499009
C	0.342381	0.224752	0.057428
C	0.873905	1.434256	0.452342
H	4.133949	0.898614	−0.309064
H	0.422059	−0.945611	2.300377
Cl	3.633529	−1.656388	−1.259930
H	−1.562895	1.330277	−1.412607
C	−1.971384	0.699067	−0.639382
C	−3.338926	0.550961	−0.523427
C	−1.115961	0.018194	0.214666
C	−3.876399	−0.260099	0.456839
C	−1.650417	−0.785123	1.223486
C	−3.028566	−0.911717	1.329813
H	−3.445568	−1.521517	2.116343
H	0.235973	2.194743	0.872656
H	−4.944957	−0.367598	0.546824
S	0.500901	−2.262080	−1.021529
S	−0.674799	−1.702921	2.365815
Cl	−4.381652	1.393177	−1.610784
Cl	2.886299	3.176242	0.809543
IM27			
0	2	-	-
C	2.368436	2.332784	0.064007
C	3.317032	1.363584	−0.203988
C	2.930244	0.044629	−0.354361
C	1.597594	−0.297888	−0.241926
C	0.612817	0.677505	0.002873
C	1.031586	1.996016	0.166527
H	4.358718	1.626830	−0.286450
H	0.429461	−1.032127	2.355218
Cl	4.100886	−1.171718	−0.686203
H	−2.007486	1.755939	−0.348142
C	−1.918776	0.688627	−0.254701
C	−3.038748	−0.104146	−0.477267
C	−0.707499	0.095579	−0.003682
C	−2.946873	−1.512956	−0.572695
C	−0.636992	−1.375057	0.236619
C	−1.782498	−2.136528	−0.305402

Table S2. *Cont.*

IM	*x*	*y*	*z*
IM27			
H	2.665793	3.359067	0.201529
H	−3.811715	−2.073012	−0.889993
S	−0.712621	−1.675768	2.087742
S	0.974265	−1.914178	−0.415481
Cl	−0.083839	3.255770	0.540424
Cl	−4.551556	0.644063	−0.780244
2,7-DCTA			
0	1	-	-
C	−1.244530	−0.954932	0.000000
C	−2.385756	−1.749032	0.000000
C	−2.312304	−3.125603	0.000000
C	−1.065834	−3.722123	0.000000
C	0.079507	−2.955935	0.000000
C	0.000028	−1.568253	0.000000
H	−3.207206	−3.725187	0.000000
C	0.000000	1.568089	0.000000
C	−0.079674	2.955947	0.000000
C	1.065490	3.722054	0.000000
C	2.312221	3.125691	0.000000
C	2.385756	1.749287	0.000000
C	1.244495	0.954950	0.000000
H	−1.044682	3.438455	0.000000
H	3.355095	1.273072	0.000000
H	1.044430	−3.438651	0.000000
H	3.206903	3.725584	0.000000
S	1.563147	−0.770676	0.000000
S	−1.562923	0.770577	0.000000
H	−3.355134	−1.272755	0.000000
Cl	0.935957	5.441618	0.000000
Cl	−0.935920	−5.441604	0.000000
2,8-DCTA			
0	1	-	-
C	−1.406782	−1.048056	0.000000
C	−2.623784	−1.719923	0.000000
C	−3.826097	−1.045811	0.000000
C	−3.810129	0.336104	0.000000
C	−2.616447	1.024375	0.000000
C	−1.406960	0.339141	0.000000
C	1.406532	−1.047578	0.000000
C	2.623499	−1.719750	0.000000
C	3.825901	−1.046208	0.000000
C	3.810095	0.335990	0.000000
C	2.616719	1.024475	0.000000

Table S2. *Cont.*

IM	*x*	*y*	*z*
2,8-DCTA			
C	1.406918	0.339439	0.000000
H	2.622914	2.103431	0.000000
H	−2.622559	2.103351	0.000000
S	0.000000	1.388816	0.000000
S	−0.000031	−2.096858	0.000000
H	−2.625395	−2.799912	0.000000
H	4.759488	−1.583404	0.000000
H	−4.759623	−1.583188	0.000000
H	2.624700	−2.799778	0.000000
Cl	5.294807	1.213046	0.000000
Cl	−5.294560	1.213718	0.000000
1,3,7-TCTA			
0	1	-	-
C	0.248717	−1.797218	0.000000
C	0.288735	−3.187123	0.000000
C	1.480850	−3.879438	0.000000
C	2.662721	−3.163685	0.000000
C	2.649010	−1.785419	0.000000
C	1.442926	−1.095012	0.000000
C	−1.195427	0.609800	0.000000
C	−2.379933	1.346322	0.000000
C	−2.402595	2.726333	0.000000
C	−1.199849	3.399787	0.000000
C	−0.007504	2.710102	0.000000
C	0.000000	1.321488	0.000000
H	0.924954	3.252240	0.000000
H	3.581046	−1.242040	0.000000
S	1.620187	0.649009	0.000000
S	−1.377984	−1.134886	0.000000
H	−0.640436	−3.737821	0.000000
H	−3.340123	3.254815	0.000000
Cl	−3.891530	0.519647	0.000000
Cl	−1.187812	5.121537	0.000000
H	1.492425	−4.956486	0.000000
Cl	4.172341	−3.996025	0.000000
1,3,8-TCTA			
0	1	-	-
C	−0.919067	−1.450979	0.000000
C	−1.967239	−2.364387	0.000000
C	−1.714175	−3.719290	0.000000
C	−0.416256	−4.193854	0.000000
C	0.619174	−3.284359	0.000000
C	0.386698	−1.913917	0.000000

Table S2. *Cont.*

IM	*x*	*y*	*z*
1,3,8-TCTA			
C	0.000000	1.200888	0.000000
C	−0.196162	2.582013	0.000000
C	0.846459	3.485795	0.000000
C	2.135098	2.997088	0.000000
C	2.372445	1.640015	0.000000
C	1.313684	0.742151	0.000000
H	3.387030	1.273813	0.000000
H	1.637319	−3.644469	0.000000
S	1.837796	−0.931444	0.000000
S	−1.455742	0.222085	0.000000
H	−2.987112	−2.011816	0.000000
H	0.648332	4.543760	0.000000
H	−0.220398	−5.253048	0.000000
Cl	−1.800789	3.210885	0.000000
Cl	3.462374	4.094085	0.000000
Cl	−3.034643	−4.827645	0.000000
1,3,6,8-TeCTA			
0	1	-	-
C	−1.249732	−0.951135	0.000000
C	−2.370723	−1.780460	0.000000
C	−2.279389	−3.156958	0.000000
C	−1.025287	−3.729382	0.000000
C	0.106627	−2.943379	0.000000
C	0.000000	−1.559575	0.000000
C	−0.000060	1.559685	0.000000
C	−0.106650	2.943603	0.000000
C	1.025285	3.729427	0.000000
C	2.279421	3.156867	0.000000
C	2.370718	1.780430	0.000000
C	1.249549	0.951298	0.000000
H	1.080465	−3.407320	0.000000
S	1.569322	−0.769931	0.000000
S	−1.569377	0.770087	0.000000
H	−3.170752	−3.760351	0.000000
H	−1.080498	3.407514	0.000000
H	3.170745	3.760318	0.000000
Cl	−3.944727	−1.079212	0.000000
Cl	−0.871971	−5.443875	0.000000
Cl	0.872395	5.443986	0.000000
Cl	3.944442	1.078796	0.000000
1,3,7,9-TeCTA			
0	1	-	-
C	0.000000	1.465637	0.000000

Table S2. *Cont.*

IM	*x*	*y*	*z*
1,3,7,9-TeCTA			
C	0.249533	2.838260	0.000000
C	−0.758355	3.780881	0.000000
C	−2.065197	3.344123	0.000000
C	−2.355194	1.996919	0.000000
C	−1.329908	1.061363	0.000000
C	0.833073	−1.207996	0.000000
C	1.814987	−2.199809	0.000000
C	1.515113	−3.546998	0.000000
C	0.189638	−3.924227	0.000000
C	−0.810341	−2.976527	0.000000
C	−0.492329	−1.625667	0.000000
H	−3.382985	1.669503	0.000000
S	−1.907510	−0.592119	0.000000
S	1.428613	0.443487	0.000000
H	−0.519422	4.830408	0.000000
Cl	1.874653	3.411003	0.000000
Cl	−3.348534	4.491040	0.000000
H	2.305296	−4.277895	0.000000
H	−1.843200	−3.287219	0.000000
Cl	3.479062	−1.752286	0.000000
Cl	−0.219502	−5.596371	0.000000
2,4,8-TCDT			
0	1	-	-
C	−2.284368	1.627546	0.000045
C	−3.073691	0.482654	−0.000067
C	−2.464923	−0.752397	−0.000131
C	−1.082030	−0.845378	−0.000070
C	−0.302214	0.314135	0.000043
C	−0.910057	1.563167	0.000099
H	−4.147875	0.558547	−0.000100
Cl	−3.414730	−2.186482	−0.000230
H	4.690107	−1.409280	−0.000021
C	3.680377	−1.032782	−0.000016
2,4,8-TCDT			
C	3.460500	0.341720	0.000080
C	2.611118	−1.901330	−0.000081
C	2.190144	0.868885	0.000115
C	1.324970	−1.381969	−0.000048
C	1.106058	−0.001845	0.000053
H	−0.326143	2.468976	0.000186
H	2.047955	1.937792	0.000187
S	−0.148873	−2.304643	−0.000072

Table S2. *Cont.*

IM	*x*	*y*	*z*
2,4,8-TCDT			
Cl	−3.064136	3.166737	0.000118
Cl	4.820184	1.407432	0.000164
H	2.780223	−2.966864	−0.000125
2,4,6,8-TeCDT			
0	1	-	-
C	−2.939152	1.382422	0.000122
C	−3.459940	0.092957	0.000066
C	−2.598510	−0.981300	−0.000014
C	−1.227577	−0.772485	−0.000084
C	−0.721321	0.529587	−0.000019
C	−1.583947	1.618138	0.000096
H	−4.525117	−0.065816	0.000059
Cl	−3.217671	−2.585268	−0.000172
H	4.525122	−0.065810	0.000128
C	3.459948	0.092964	0.000105
C	2.939154	1.382421	0.000115
C	2.598507	−0.981300	0.000035
C	1.583941	1.618140	0.000067
C	1.227578	−0.772482	−0.000046
Cl	3.217692	−2.585268	−0.000046
C	0.721313	0.529586	−0.000005
H	−1.210427	2.629011	0.000216
H	1.210430	2.629014	0.000144
S	−0.000007	−1.998115	−0.000447
Cl	−4.034365	2.715011	0.000214
Cl	4.034353	2.715027	0.000237

Table S3. Cartesian coordinates for the transition states involved in the formation of PCTA/DTs from the 2,4-DCTP and 2,4,6-TCTP as precursors.

TS	*x*	*y*	*z*
**TS1**			
0	2	-	-
C	1.161015	0.116852	−0.956665
C	1.768908	−1.065374	−0.531835
C	3.098275	−1.083787	−0.152171
C	3.821789	0.091672	−0.188986
C	3.248202	1.279246	−0.607427

Table S3. *Cont.*

TS	*x*	*y*	*z*
**TS1**			
C	1.924379	1.278729	−0.991214
Cl	0.893256	−2.542967	−0.464166
H	3.556493	−2.002026	0.173724
H	1.460320	2.192739	−1.324721
C	−1.439683	0.726630	−0.046237
C	−2.847254	0.681467	−0.487677
C	−3.667349	−0.263249	−0.024982
C	−3.225761	−1.245776	0.929202
C	−1.996978	−1.171340	1.465308
C	−1.086922	−0.096051	1.157230
Cl	−1.056970	2.562520	0.297877
H	−3.159742	1.396287	−1.231004
H	−1.674781	−1.876479	2.214156
H	−0.607610	4.248418	0.767465
H	−3.910826	−2.026035	1.219452
H	3.831651	2.184416	−0.632907
S	−0.505482	0.154347	−1.505781
S	0.237984	0.160770	2.063665
Cl	−5.298301	−0.361807	−0.564042
Cl	5.475707	0.068530	0.289179
TS2			
0	2	-	-
C	1.340277	−0.173186	−0.830866
C	2.240737	−1.191894	−0.526628
C	3.538524	−0.902400	−0.153465
C	3.938059	0.418670	−0.082120
C	3.072759	1.447565	−0.399793
C	1.784710	1.142253	−0.791070
Cl	1.771846	−2.845398	−0.579272
H	4.223275	−1.698052	0.086247
H	1.110669	1.941801	−1.049789
C	−1.469991	−0.121740	−0.203481
C	−2.767824	−0.080872	−0.836076
C	−3.895463	−0.238884	−0.113656
C	−3.856586	−0.474040	1.289589
C	−2.666199	−0.609029	1.910605
C	−1.417025	−0.567440	1.206011
Cl	−1.201105	1.952657	0.296179
H	−2.812206	0.129137	−1.892522
H	−2.615469	−0.811358	2.967906
O	−0.402114	3.954986	0.039581
H	−1.105441	4.262015	−0.544632
H	−4.782616	−0.551708	1.835317

Table S3. *Cont.*

TS	*x*	*y*	*z*
TS2			
H	3.400609	2.472383	−0.350574
S	−0.276857	−0.541362	−1.436545
S	−0.038778	−1.015226	1.981644
Cl	−5.430106	−0.141522	−0.873837
Cl	5.553270	0.777044	0.397842
TS3			
0	2	-	-
C	1.337802	−0.341897	−0.862469
C	2.188731	−1.396069	−0.539388
C	3.496865	−1.160926	−0.164811
C	3.955004	0.141794	−0.108060
C	3.137733	1.205671	−0.439893
C	1.838694	0.952784	−0.830966
Cl	1.639632	−3.025718	−0.558254
H	4.144186	−1.982813	0.089926
H	1.196822	1.776295	−1.099500
C	−1.452125	−0.190768	−0.174021
C	−2.765342	−0.184819	−0.802703
C	−3.870378	−0.498663	−0.106867
C	−3.806608	−0.883087	1.266602
C	−2.611981	−0.977998	1.882988
C	−1.366900	−0.724138	1.215799
Cl	−1.224883	1.811430	0.281067
H	−2.824779	0.116944	−1.835953
H	−2.547891	−1.279349	2.915766
H	−1.087795	4.417239	−0.931680
H	−4.722574	−1.099610	1.791807
H	3.512039	2.215057	−0.401677
S	−0.298550	−0.637696	−1.448559
S	0.040639	−0.974800	2.015992
Cl	−5.414334	−0.447086	−0.854844
Cl	5.582960	0.433165	0.373308
S	−0.246863	4.179028	0.079587
TS4			
0	2	-	-
C	1.297590	−0.416310	−0.893681
C	2.110470	−1.474920	−0.495034
C	3.415835	−1.252465	−0.101554
C	3.906143	0.039454	−0.103260
C	3.125173	1.105693	−0.511500
C	1.830053	0.866637	−0.918407
Cl	1.520194	−3.090602	−0.447665
H	4.036406	−2.075422	0.210012

Table S3. *Cont.*

TS	*x*	*y*	*z*
TS4			
H	1.216199	1.691345	−1.242633
C	−1.451900	−0.137552	−0.204684
C	−2.786062	−0.184006	−0.770515
C	−3.852268	−0.523670	−0.017289
C	−3.708599	−0.895539	1.348455
C	−2.484040	−0.907944	1.921740
C	−1.292191	−0.537296	1.222718
Cl	−1.313652	1.924547	−0.027839
H	−2.899484	0.074873	−1.810920
H	−2.366315	−1.181548	2.957546
Cl	−0.090117	4.052595	−0.015247
H	−4.586859	−1.163111	1.913207
H	3.524762	2.105934	−0.515824
S	−0.342605	−0.694482	−1.467698
S	0.132890	−0.477426	2.034444
Cl	−5.423440	−0.542630	−0.699608
Cl	5.528751	0.315266	0.399697
TS5			
0	2	-	-
C	−1.145094	1.135455	−0.039942
C	−1.373409	−0.143005	0.566004
C	−2.712916	−0.637298	0.609857
C	−3.707125	0.033509	−0.030531
C	−3.469378	1.261069	−0.666289
C	−2.197749	1.788077	−0.664202
Cl	−0.411115	−0.576003	1.952088
H	−2.902665	−1.559354	1.132457
H	−2.001998	2.717694	−1.175164
C	1.546839	0.533162	−0.257736
C	2.864654	0.831933	0.066112
C	3.846865	−0.120306	−0.099001
C	3.534102	−1.393296	−0.541391
C	2.218050	−1.697837	−0.813294
C	1.205284	−0.746105	−0.703197
H	3.122980	1.809554	0.441754
H	1.955635	−2.697786	−1.121610
H	4.305540	−2.138098	−0.646446
H	−4.279084	1.776178	−1.155494
S	0.425742	1.876604	−0.099009
S	−0.416262	−1.230936	−1.118354
Cl	−5.308093	−0.602273	−0.027503
Cl	5.483160	0.277628	0.278000

Table S3. *Cont.*

TS	*x*	*y*	*z*
TS6			
0	2	-	-
C	−1.031526	0.656093	−0.186831
C	−2.259364	1.254531	0.046220
C	−3.442209	0.550825	−0.081842
C	−3.405753	−0.789831	−0.476793
C	−2.224659	−1.417914	−0.735973
C	−0.997370	−0.732788	−0.517162
Cl	−2.331674	2.913301	0.500904
H	−4.382880	1.037496	0.112673
H	−0.146708	−1.073582	−1.091188
C	1.673146	0.304723	0.113458
C	2.912121	0.675624	−0.395143
C	3.993975	−0.167234	−0.249475
C	3.854633	−1.398196	0.366287
C	2.611340	−1.781817	0.825910
C	1.505435	−0.941948	0.729414
H	3.030884	1.623962	−0.894647
H	2.484545	−2.755066	1.273639
H	4.702950	−2.055971	0.461234
H	−2.203933	−2.441171	−1.071599
S	0.434003	1.552784	0.062124
S	−0.043696	−1.476218	1.325205
Cl	−4.898093	−1.629099	−0.671777
Cl	5.531627	0.313849	−0.865349
TS7			
0	2	-	-
C	−1.037110	0.555525	0.393026
C	−2.113806	1.328305	−0.013405
C	−3.303670	0.754028	−0.428623
C	−3.420532	−0.625526	−0.433589
C	−2.370953	−1.432520	−0.065460
C	−1.145002	−0.846390	0.281051
Cl	−2.023348	3.046835	0.037264
H	−4.128546	1.378146	−0.727058
H	−0.361581	−0.958084	−1.323217
C	1.661523	0.196359	0.431924
C	2.840794	0.745649	−0.047759
C	3.878477	−0.091622	−0.409322
C	3.746619	−1.466732	−0.339499
C	2.552865	−2.007226	0.097536
C	1.514759	−1.183872	0.504196
H	2.949968	1.814706	−0.132018
H	2.424961	−3.077878	0.127720

Table S3. *Cont.*

TS	*x*	*y*	*z*
TS7			
H	4.561830	−2.103868	−0.640305
H	−2.466031	−2.505424	−0.082764
S	0.419213	1.296685	1.019890
S	0.026134	−1.875192	1.126306
Cl	−4.916038	−1.338000	−0.906049
Cl	5.355272	0.594262	−0.977213
TS8			
0	2	-	-
C	−0.942353	0.310098	−0.288064
C	−1.080586	−1.069957	−0.659132
C	−2.281417	−1.715738	−0.629935
C	−3.433286	−0.996595	−0.314539
C	−3.379703	0.370024	−0.065683
C	−2.175446	1.022498	−0.102264
H	−2.349129	−2.765180	−0.864223
C	1.470101	−0.137463	1.040276
C	2.501138	−0.776557	1.714657
C	3.757264	−0.884046	1.144961
C	3.974289	−0.379664	−0.123276
C	2.955624	0.228501	−0.833989
C	1.713421	0.361799	−0.240272
H	2.323804	−1.184127	2.697354
H	−4.282952	0.920740	0.136915
H	4.559040	−1.369225	1.677003
H	3.132557	0.591795	−1.833392
H	−0.179758	−1.604663	−0.918906
Cl	−2.142108	2.706601	0.195879
S	0.409489	1.213165	−1.041942
S	−0.108400	0.099708	1.730261
Cl	−4.949351	−1.795446	−0.272939
Cl	5.532914	−0.534256	−0.850252
TS9			
0	2	-	-
C	−1.002121	0.457971	0.316354
C	−1.043842	−0.833236	0.942377
C	−2.139986	−1.641189	0.875775
C	−3.294808	−1.170362	0.252158
C	−3.355341	0.118085	−0.265350
C	−2.258100	0.936238	−0.188728
H	−2.127152	−2.626215	1.312011
C	1.592084	0.806519	0.675089
C	2.715505	0.891409	1.476744

Table S3. *Cont.*

TS	*x*	*y*	*z*
TS9			
C	3.887404	0.256890	1.102658
C	3.905779	−0.486935	−0.062479
C	2.782440	−0.611195	−0.859022
C	1.618456	0.053446	−0.501828
H	2.680021	1.453790	2.396829
H	−4.266147	0.482440	−0.710297
H	2.815417	−1.217264	−1.749435
Cl	−2.365067	2.522208	−0.820723
H	−0.144758	−1.172290	1.433944
H	4.769589	0.323602	1.717429
S	0.107651	1.660256	1.048633
S	0.177070	−0.007341	−1.471863
Cl	−4.678907	−2.176748	0.153957
Cl	5.354131	−1.307610	−0.522133
TS10			
0	2	-	-
C	1.188801	0.706810	−0.272705
C	2.485921	1.060936	0.065750
C	3.510972	0.135051	0.064655
C	3.243837	−1.186507	−0.307318
C	1.989544	−1.579901	−0.665323
C	0.908624	−0.659764	−0.576056
Cl	2.844595	2.689327	0.493901
H	4.508407	0.434024	0.339013
C	−1.682084	−0.325652	0.456603
C	−2.934127	−0.932038	0.496453
C	−4.027047	−0.313547	−0.074881
C	−3.898968	0.891559	−0.742082
C	−2.653275	1.479399	−0.816915
C	−1.546623	0.900599	−0.206839
H	−3.045077	−1.891002	0.975886
H	1.795972	−2.590924	−0.982230
H	0.058658	−0.849332	−1.217162
H	−2.536179	2.412063	−1.347331
S	−0.323201	−1.134057	1.192465
S	−0.086150	1.879937	−0.182254
Cl	4.550900	−2.308603	−0.345198
H	−4.755921	1.352534	−1.204394
Cl	−5.571361	−1.078555	0.012755
TS11			
0	2	-	-
C	1.178627	0.657351	0.293958
C	2.410894	1.095018	−0.165846

Table S3. *Cont.*

TS	*x*	*y*	*z*
TS11			
C	3.433785	0.207828	−0.456199
C	3.221116	−1.148676	−0.277771
C	2.008091	−1.631165	0.151205
C	0.953694	−0.733052	0.370646
Cl	2.728992	2.777812	−0.340661
H	4.385367	0.575339	−0.800429
H	0.175004	−0.876303	−1.233823
C	−1.713270	−0.412496	0.537620
C	−2.908133	−1.033589	0.211921
C	−3.929859	−0.283577	−0.337495
C	−3.762493	1.062072	−0.608972
C	−2.553182	1.663967	−0.318900
C	−1.529529	0.939957	0.274567
H	−3.039529	−2.088842	0.387324
H	−2.403808	2.707629	−0.547451
H	1.846476	−2.688764	0.277289
S	−0.430997	−1.338690	1.299224
S	−0.069222	1.792150	0.761647
Cl	4.508908	−2.247800	−0.595953
H	−4.564388	1.627261	−1.054525
Cl	−5.429315	−1.048454	−0.713429
TS12			
0	2	-	-
C	1.164953	0.773927	−0.286347
C	1.564424	−0.006883	0.853776
C	2.797916	−0.604161	0.921458
C	3.733642	−0.353027	−0.070815
C	3.455505	0.530787	−1.117213
C	2.224150	1.108262	−1.196503
Cl	0.451105	−0.280327	2.120732
H	3.041972	−1.245539	1.751585
C	−1.502187	−0.127047	−0.688199
C	−2.654486	−0.900600	−0.698361
C	−3.802986	−0.411860	−0.105351
C	−3.822834	0.816526	0.527771
C	−2.663644	1.571239	0.565324
C	−1.514624	1.111627	−0.050406
H	−2.658816	−1.871395	−1.165956
H	4.217253	0.756707	−1.844954
H	1.987104	1.787654	−1.999318
H	−2.657743	2.518956	1.081034
H	−4.724995	1.169656	0.998822
S	−0.035272	−0.657275	−1.454785

Table S3. *Cont.*

TS	*x*	*y*	*z*
TS12			
S	−0.049263	2.072546	−0.092708
Cl	5.273364	−1.100077	0.018125
Cl	−5.238067	−1.373309	−0.138393
TS13			
0	2	-	-
C	−1.320609	1.199234	−0.092161
C	−2.442103	1.600340	−0.804550
C	−3.588688	0.839117	−0.828045
C	−3.625374	−0.373735	−0.123919
C	−2.558804	−0.804762	0.599523
C	−1.336969	−0.064579	0.582190
H	−4.452440	1.160755	−1.385474
C	1.377264	−0.233035	−0.514029
C	2.546530	−0.991403	−0.512147
C	3.753209	−0.414089	−0.183785
C	3.825412	0.913247	0.199601
C	2.665082	1.656580	0.242213
C	1.444132	1.109653	−0.133498
H	2.500295	−2.037806	−0.766662
H	−2.598311	−1.715719	1.172167
H	2.706811	2.685178	0.567421
Cl	−0.392827	−0.235984	2.037289
H	−2.397875	2.520221	−1.366247
H	4.770427	1.350379	0.476176
S	0.083967	2.221054	−0.127755
S	−0.095819	−1.039060	−0.981778
Cl	−5.076499	−1.302665	−0.147414
Cl	5.188516	−1.372781	−0.215359
TS14			
0	2	-	-
C	1.640357	0.341541	0.287074
C	2.843174	1.027642	0.450726
C	4.034247	0.525532	−0.034826
C	4.034764	−0.683367	−0.701818
C	2.863848	−1.396015	−0.866794
C	1.683578	−0.886904	−0.360596
Cl	2.880402	2.536804	1.282412
H	4.947441	1.079281	0.104343
H	0.786058	−1.477536	−0.444507
C	−1.158889	0.366314	−0.027597
C	−2.077354	1.448120	−0.436552
C	−3.356889	1.174060	−0.697154
C	−3.884170	−0.148103	−0.530649

Table S3. *Cont.*

TS	*x*	*y*	*z*
TS14			
C	−3.137566	−1.130291	0.013750
C	−1.788069	−0.895738	0.493285
H	−4.904475	−0.342437	−0.818501
H	−0.672683	0.039615	−1.035393
Cl	−3.853282	−2.668780	0.253463
H	2.873766	−2.348685	−1.369850
H	−1.663045	2.428967	−0.601051
S	0.184507	1.073935	0.944527
S	−1.007378	−1.929127	1.475455
Cl	5.518001	−1.307603	−1.319984
Cl	−4.438392	2.384906	−1.267141
H	−0.197730	−0.223434	−2.216616
TS15			
0	2	-	-
C	−1.308504	−0.823432	−0.809260
C	−1.836336	0.451134	−1.015939
C	−3.144465	0.742430	−0.670925
C	−3.924439	−0.246682	−0.106317
C	−3.427911	−1.520043	0.113885
C	−2.125637	−1.796677	−0.243101
Cl	−0.885281	1.705197	−1.703629
H	−3.541087	1.730013	−0.834635
H	−1.715094	−2.779122	−0.073123
C	1.261645	−1.090553	0.288808
C	2.631169	−1.498189	−0.047641
C	3.578477	−0.564491	−0.165478
C	3.295087	0.827123	0.054576
C	2.097289	1.229600	0.522206
C	1.074132	0.267047	0.874788
H	2.822287	−2.537308	−0.260587
O	0.121512	−2.668780	1.955772
H	−0.391184	−1.945490	2.347554
H	4.071778	1.550079	−0.134119
H	−4.054165	−2.278074	0.554090
S	0.327230	−1.240397	−1.289672
S	−0.196431	0.612042	1.831636
Cl	5.189649	−0.969067	−0.611574
Cl	−5.550236	0.117681	0.327790
H	0.788682	−1.881661	1.002694
Cl	1.830036	2.892180	0.828997
TS16			
0	2	-	-
C	−1.686256	0.541124	−0.246353

Table S3. *Cont.*

TS	*x*	*y*	*z*
TS16			
C	−2.915801	1.173402	−0.424497
C	−4.104267	0.555840	−0.091364
C	−4.073577	−0.718927	0.437558
C	−2.872110	−1.373889	0.621706
C	−1.692977	−0.746885	0.269894
Cl	−2.981449	2.768220	−1.076412
H	−5.038484	1.070301	−0.241206
H	−0.772890	−1.290442	0.399236
C	1.144896	0.526119	−0.104280
C	2.200597	1.464469	0.298711
C	3.462720	1.029324	0.402744
C	3.814221	−0.317475	0.071403
C	2.918029	−1.139212	−0.517294
C	1.588715	−0.677620	−0.879017
H	4.818854	−0.658767	0.260980
H	0.827808	0.053951	0.953406
Cl	3.408590	−2.713113	−0.970303
H	2.123980	−0.720820	2.581465
H	−2.853273	−2.371945	1.026976
H	1.915540	2.455304	0.612255
S	−0.259346	1.464497	−0.691436
S	0.674169	−1.408359	−2.003737
Cl	−5.552502	−1.495007	0.861579
Cl	4.717182	2.053798	0.974962
S	0.785950	−0.735606	2.629210
TS17			
0	2	-	-
C	−1.582618	−0.164214	0.395382
C	−2.785284	−0.683060	0.864146
C	−3.988999	−0.282027	0.318293
C	−3.985654	0.642165	−0.706230
C	−2.799734	1.174947	−1.185767
C	−1.608876	0.771776	−0.630568
Cl	−2.807949	−1.837427	2.138094
H	−4.912384	−0.693522	0.688975
H	−0.689082	1.189405	−1.005770
C	1.257969	−0.303555	0.081369
C	2.274995	−1.356385	−0.062148
C	3.535570	−0.985003	−0.326097
C	3.908072	0.393830	−0.382172
C	3.038056	1.380014	−0.053210
C	1.734174	1.040502	0.407135
H	4.922359	0.645410	−0.649535
H	0.754411	−0.411299	−0.947363

Table S3. *Cont.*

TS	*x*	*y*	*z*
TS17			
Cl	3.552392	3.012203	0.000476
H	−2.813651	1.893436	−1.987811
H	1.943266	−2.379722	−0.121133
S	−0.121566	−0.706456	1.186781
S	0.778617	1.983081	1.363773
Cl	−5.479918	1.142336	−1.391317
Cl	4.761553	−2.144855	−0.651623
Cl	−0.243948	−1.963059	−1.807808
TS18			
0	2	-	-
C	−1.421946	−1.289928	−0.338548
C	−1.570546	−0.223583	0.605671
C	−2.871752	0.339270	0.783250
C	−3.895461	−0.045944	−0.023832
C	−3.731104	−1.055566	−0.984624
C	−2.501028	−1.655871	−1.129489
Cl	−0.618608	−0.277314	2.064498
H	−3.007707	1.086851	1.546145
H	−2.358696	−2.412402	−1.885170
C	1.312738	−0.847729	−0.347508
C	2.583469	−1.346801	−0.095957
C	3.647290	−0.480953	0.019185
C	3.464811	0.884505	−0.079543
C	2.190749	1.367981	−0.288558
C	1.079714	0.530012	−0.433386
H	4.294098	1.564099	0.017455
Cl	1.988107	3.074495	−0.395015
H	−4.563360	−1.346294	−1.603465
H	2.739803	−2.408990	0.003808
S	0.096674	−2.091099	−0.596982
S	−0.495358	1.227043	−0.678696
Cl	5.227990	−1.105659	0.302454
Cl	−5.447299	0.678434	0.158187
TS19			
0	2	-	-
C	1.365953	−0.851736	0.132368
C	2.633039	−1.264857	−0.247503
C	3.744691	−0.476452	−0.023259
C	3.594515	0.757777	0.617922
C	2.371164	1.200859	1.022689
C	1.208537	0.440053	0.717745
Cl	2.843071	−2.793704	−1.009420
H	4.718674	−0.816123	−0.332194

Table S3. *Cont.*

TS	*x*	*y*	*z*
TS19			
H	0.356650	0.579163	1.369190
C	−1.372813	−0.739497	−0.026346
C	−2.535234	−1.339041	0.439446
C	−3.711848	−0.621024	0.459900
C	−3.745080	0.696283	0.048544
C	−2.574386	1.292863	−0.375700
C	−1.359839	0.604599	−0.431894
H	−4.662405	1.259685	0.070535
Cl	−2.649786	2.939796	−0.869694
H	2.266321	2.136910	1.545051
H	−2.520774	−2.364163	0.772440
S	0.104800	1.400418	−0.930631
S	−0.014525	−1.837672	−0.230672
Cl	5.005356	1.696720	0.928560
Cl	−5.158350	−1.372129	1.018339
TS20			
0	2	-	-
C	1.364064	0.717251	0.404906
C	2.532762	1.356598	0.023529
C	3.637808	0.643301	−0.409952
C	3.571321	−0.738913	−0.460850
C	2.423987	−1.413233	−0.117252
C	1.286498	−0.681198	0.251993
Cl	2.663170	3.069264	0.129360
H	4.539225	1.161873	−0.688546
H	0.487768	−0.642721	−1.349203
C	−1.358348	0.691191	0.413447
C	−2.439237	1.411926	−0.067002
C	−3.581251	0.734627	−0.445828
C	−3.651589	−0.642811	−0.383037
C	−2.551119	−1.349919	0.063092
C	−1.393279	−0.699496	0.475675
H	−4.543629	−1.164210	−0.685901
Cl	−2.656542	−3.065597	0.122842
H	2.376525	−2.488146	−0.170138
H	−2.391957	2.486057	−0.137278
S	−0.001773	−1.582181	1.073800
S	0.003803	1.611968	1.044956
Cl	−4.941644	1.621557	−1.019404
Cl	4.961480	−1.625541	−0.959936
TS21			
0	2	-	-

Table S3. *Cont.*

TS	*x*	*y*	*z*
TS21			
C	−1.349101	−0.563909	−0.668472
C	−1.781986	−0.627016	0.700094
C	−3.070998	−0.326401	1.061364
C	−4.014461	−0.069741	0.076227
C	−3.681977	−0.144762	−1.278388
C	−2.397366	−0.423187	−1.637693
Cl	−0.645136	−0.992733	1.921068
H	−3.350424	−0.310632	2.101442
C	1.211324	0.664648	−0.434977
C	2.318320	1.380767	0.002029
C	3.536739	0.768360	0.232251
C	3.645764	−0.593611	0.039644
C	2.563407	−1.348179	−0.373656
C	1.363885	−0.709720	−0.619519
H	−4.442625	0.005753	−2.026559
H	−2.119122	−0.481969	−2.677558
H	4.380118	1.349386	0.564637
H	2.657425	−2.415058	−0.493007
Cl	2.186593	3.080301	0.243865
S	−0.328104	1.396483	−0.757105
S	−0.009645	−1.610406	−1.225109
Cl	5.154998	−1.372873	0.340436
Cl	−5.624849	0.298939	0.533045
TS22			
0	2	-	-
C	−1.224490	0.382396	−0.009348
C	−1.335633	−0.500030	−1.136143
C	−2.501350	−1.131512	−1.454006
C	−3.651389	−0.846686	−0.718994
C	−3.634161	0.092466	0.305671
C	−2.464612	0.732318	0.623962
H	−2.545483	−1.829523	−2.273481
C	1.297321	−0.587469	0.664934
C	2.385251	−1.439508	0.772583
C	3.587773	−1.075139	0.196348
C	3.726798	0.104639	−0.506980
C	2.626902	0.934337	−0.629897
C	1.411334	0.609448	−0.050265
H	2.299163	−2.371991	1.304981
H	−4.538959	0.330816	0.839296
Cl	−2.471070	1.883609	1.888165
H	−0.439176	−0.690162	−1.706364

Table S3. *Cont.*

TS	*x*	*y*	*z*
TS22			
H	4.665281	0.373959	−0.960166
Cl	2.795040	2.407302	−1.506570
S	0.020335	1.665695	−0.120624
S	−0.223284	−0.942417	1.428050
Cl	−5.123295	−1.633894	−1.105693
Cl	4.944259	−2.129787	0.343493
TS23			
0	2	-	-
C	1.363788	0.586445	−0.109449
C	2.626147	1.015494	0.275012
C	3.738360	0.213810	0.115433
C	3.599598	−1.050218	−0.467806
C	2.383709	−1.510268	−0.875251
C	1.218656	−0.734113	−0.628412
Cl	2.829938	2.581162	0.959392
H	4.706458	0.566420	0.428470
C	−1.347667	−0.805645	0.517317
C	−2.515151	−1.560811	0.520124
C	−3.702846	−1.001880	0.100395
C	−3.753188	0.295392	−0.368567
C	−2.587277	1.033682	−0.400540
C	−1.370072	0.516852	0.045585
H	−2.484254	−2.585604	0.851374
H	2.286254	−2.471018	−1.352229
H	0.369937	−0.914893	−1.273318
H	−4.678145	0.728179	−0.709096
Cl	−2.688245	2.653510	−0.980268
S	−0.006674	1.619647	0.144511
S	0.112665	−1.567508	1.090821
Cl	5.014071	−2.005570	−0.701493
Cl	−5.145202	−1.943792	0.130984
TS24			
0	2	-	-
C	1.356087	0.488925	0.361221
C	2.524091	1.083498	−0.091232
C	3.629584	0.328140	−0.445456
C	3.567912	−1.051216	−0.340866
C	2.423428	−1.685280	0.079440
C	1.284856	−0.918060	0.363944
Cl	2.656419	2.796755	−0.175484
H	4.528602	0.814856	−0.782940
H	0.496362	−1.063270	−1.233330

Table S3. *Cont.*

TS	*x*	*y*	*z*
TS24			
C	−1.394263	−0.893021	0.572093
C	−2.501677	−1.644932	0.221663
C	−3.615923	−1.000909	−0.277805
C	−3.625409	0.366618	−0.465449
C	−2.498255	1.098582	−0.142011
C	−1.365964	0.487825	0.391412
H	−2.492967	−2.715898	0.338537
H	2.378529	−2.759367	0.148542
H	−4.494119	0.862124	−0.864248
Cl	−2.547604	2.800317	−0.388778
S	0.005824	1.462584	0.904198
S	−0.009629	−1.706140	1.281794
Cl	−5.013306	−1.918657	−0.691640
Cl	4.959650	−1.984449	−0.740812
TS25			
0	2	-	-
C	−1.374339	0.464044	−0.519333
C	−2.421228	0.609632	−1.489102
C	−3.703423	0.227508	−1.231889
C	−4.034596	−0.250942	0.038030
C	−3.093811	−0.292318	1.056904
C	−1.807335	0.113879	0.804852
H	−4.463252	0.301284	−1.992203
C	1.190898	−0.768645	−0.630883
C	2.265003	−1.616046	−0.404251
C	3.478155	−1.078869	−0.018603
C	3.642600	0.278618	0.169432
C	2.555644	1.109024	−0.034178
C	1.329803	0.605856	−0.434172
H	2.159511	−2.680525	−0.531356
H	−3.373444	−0.617719	2.044806
Cl	−0.677683	0.117051	2.085655
H	−2.143141	0.978114	−2.463323
H	4.589972	0.687015	0.476626
Cl	2.751123	2.804136	0.203948
S	−0.045931	1.643935	−0.732848
S	−0.340293	−1.376531	−1.181851
Cl	4.817738	−2.132144	0.248419
Cl	−5.641431	−0.751223	0.359614
TS26			
0	2	-	-
C	−1.461418	1.041575	−0.235537
C	−2.532400	1.481713	−0.999953

Table S3. *Cont.*

TS	*x*	*y*	*z*
TS26			
C	−3.758830	0.859671	−0.941586
C	−3.931267	−0.246624	−0.095673
C	−2.917718	−0.707593	0.684047
C	−1.618896	−0.121607	0.585450
H	−4.583083	1.208318	−1.540971
C	1.042861	−0.710776	−0.498025
C	2.109410	−1.602765	−0.434827
C	3.387469	−1.145165	−0.203720
C	3.634186	0.196452	0.009398
C	2.570139	1.072360	−0.018382
C	1.266098	0.654533	−0.280538
H	1.927672	−2.656798	−0.565013
H	−3.059446	−1.532124	1.361800
Cl	−0.679376	−0.230180	2.047855
H	−2.385774	2.317836	−1.665441
H	4.630100	0.557150	0.201182
Cl	2.897958	2.744109	0.249600
S	0.039304	1.908306	−0.354315
S	−0.519829	−1.396218	−0.856431
Cl	−5.479390	−0.997768	−0.018767
Cl	4.697344	−2.264304	−0.151838
TS27			
0	2	-	-
C	1.290617	0.473249	−0.791851
C	1.650913	−0.866868	−0.986286
C	2.907853	−1.341147	−0.665434
C	3.837670	−0.459750	−0.154339
C	3.545834	0.879725	0.009903
C	2.285126	1.335658	−0.318985
Cl	0.540841	−1.993267	−1.657464
H	3.150846	−2.379219	−0.813065
C	−1.345595	0.876089	0.248315
C	−2.684015	1.235118	−0.255183
C	−3.627840	0.301496	−0.366874
C	−3.384932	−1.065417	−0.001547
C	−2.220707	−1.434086	0.563264
C	−1.176150	−0.476889	0.882471
Cl	−0.952395	2.202727	1.546485
H	−2.844169	2.255099	−0.563328
H	−0.346351	3.392753	2.788886
H	−4.159013	−1.794810	−0.173673
H	4.290084	1.564034	0.379274
S	−0.301275	1.053584	−1.244118

Table S3. *Cont.*

TS	*x*	*y*	*z*
TS27			
S	0.098284	−0.832313	1.819677
Cl	−5.189965	0.681141	−0.975901
Cl	5.402242	−1.035943	0.261058
Cl	−2.003597	−3.072497	1.009147
Cl	2.007803	3.022471	−0.172017
TS28			
0	2	-	-
C	1.577340	0.329588	−0.757094
C	1.777775	−1.050085	−0.724022
C	2.978717	−1.616665	−0.345102
C	4.035188	−0.789578	−0.025712
C	3.896299	0.582031	−0.057037
C	2.674884	1.125342	−0.403136
Cl	0.511778	−2.124698	−1.157277
H	3.084388	−2.687435	−0.310769
C	−1.284986	0.456612	−0.447278
C	−2.202601	−0.253565	−1.302730
C	−3.052102	−1.165224	−0.777322
C	−3.012871	−1.501629	0.595473
C	−2.090008	−0.939284	1.420451
C	−1.127924	0.023929	0.963464
Cl	−2.463442	2.142771	−0.254601
H	−2.200937	−0.025172	−2.355156
O	−3.008633	4.103257	0.308546
H	−2.608717	4.113157	1.186553
S	0.124858	1.161652	−1.312522
S	−0.002879	0.702926	1.944151
Cl	−4.173119	−1.989406	−1.779551
Cl	5.546893	−1.479607	0.414173
Cl	−2.092486	−1.402700	3.067068
Cl	2.542681	2.836898	−0.355983
H	−3.723394	−2.210356	0.987153
H	4.718572	1.226286	0.202959
TS29			
0	2	-	-
C	1.543276	0.364857	−0.868078
C	1.907940	−0.980617	−0.811087
C	3.148648	−1.392131	−0.366355
C	4.072005	−0.435340	0.002189
C	3.770078	0.909278	−0.062126
C	2.514357	1.296795	−0.488438
Cl	0.797025	−2.201030	−1.288669
H	3.389245	−2.440455	−0.321805

Table S3. *Cont.*

TS	*x*	*y*	*z*
TS29			
C	−1.259917	0.150376	−0.477803
C	−2.079300	−0.775542	−1.236198
C	−2.656410	−1.825530	−0.626391
C	−2.420673	−2.110196	0.745102
C	−1.588366	−1.333010	1.479839
C	−0.928744	−0.173082	0.938617
Cl	−2.608720	1.645453	−0.285916
H	−2.226751	−0.579163	−2.284855
H	−2.491055	4.493571	−0.451935
S	0.006235	0.937877	−1.501748
S	0.036249	0.804437	1.824890
Cl	−3.666409	−2.902661	−1.502816
Cl	5.626699	−0.927918	0.545088
Cl	−1.323604	−1.751066	3.118242
Cl	2.187160	2.980467	−0.527827
H	−2.901385	−2.962344	1.196041
H	4.495000	1.651326	0.225280
S	−3.393046	3.965607	0.381478
TS30			
0	2	-	-
C	1.487380	−0.052320	−0.985716
C	1.939450	−1.369632	−0.868557
C	3.204160	−1.663234	−0.399525
C	4.038681	−0.619971	−0.050633
C	3.633961	0.697110	−0.152989
C	2.363204	0.971953	−0.616174
Cl	0.909303	−2.685716	−1.262407
H	3.527906	−2.685537	−0.306936
C	−1.307558	0.241854	−0.465203
C	−2.586282	0.161130	−1.136415
C	−3.555951	−0.641427	−0.654815
C	−3.341685	−1.468071	0.478198
C	−2.158125	−1.440697	1.140134
C	−1.087236	−0.548599	0.776392
Cl	−1.601894	2.155794	0.299727
H	−2.731413	0.742449	−2.032747
Cl	−0.873154	4.275885	1.159772
H	−4.133934	−2.115658	0.814865
H	4.291361	1.499764	0.134060
S	−0.060756	0.296606	−1.729624
S	0.225041	−0.316794	1.724385
Cl	−5.080606	−0.728536	−1.432403

Table S3. *Cont.*

TS	*x*	*y*	*z*
TS30			
Cl	5.616980	−0.969543	0.529904
Cl	−1.968671	−2.458714	2.500676
Cl	1.859072	2.603383	−0.672541
TS31			
0	2	-	-
C	−1.318859	0.941359	0.168821
C	−1.298585	−0.352034	0.787793
C	−2.509368	−1.100379	0.872312
C	−3.632191	−0.639693	0.261463
C	−3.657397	0.607171	−0.374359
C	−2.513182	1.373270	−0.403350
Cl	−0.243753	−0.586153	2.154946
H	−2.498257	−2.036880	1.402899
C	1.431933	0.778107	−0.060299
C	2.659383	1.311504	0.307137
C	3.801272	0.553838	0.168932
C	3.739808	−0.743008	−0.301625
C	2.508807	−1.272911	−0.625168
C	1.322947	−0.539017	−0.522502
H	2.721582	2.315604	0.694835
H	4.630569	−1.339101	−0.402807
H	−4.561024	0.966515	−0.836063
S	0.103410	1.923367	0.023712
S	−0.195014	−1.287552	−0.913032
Cl	−5.079800	−1.567811	0.305281
Cl	5.326516	1.226574	0.601682
Cl	−2.583068	2.906566	−1.177781
Cl	2.460421	−2.896142	−1.196168
TS32			
0	2	-	-
C	−1.201802	−0.634378	0.029151
C	−1.518100	0.134967	1.207355
C	−2.752794	0.689952	1.416792
C	−3.781098	0.407266	0.529664
C	−3.587275	−0.450816	−0.547084
C	−2.352370	−0.997009	−0.762035
H	−2.925059	1.318332	2.273972
C	1.361284	0.406730	−0.621034
C	2.493961	1.203721	−0.730068
C	3.721928	0.792936	−0.244915
C	3.814832	−0.434226	0.379981
C	2.708025	−1.248474	0.531348

Table S3. *Cont.*

TS	*x*	*y*	*z*
TS32			
C	1.497591	−0.826041	0.018488
H	−4.405812	−0.696109	−1.202129
H	4.585368	1.427453	−0.350214
H	2.792688	−2.190308	1.048160
Cl	−2.157360	−2.041073	−2.101588
S	0.092449	−1.863503	0.125635
S	−0.188762	0.869495	−1.243464
Cl	−5.329024	1.092204	0.787255
Cl	5.336992	−0.952067	1.003021
Cl	−0.284958	0.450022	2.346787
Cl	2.379569	2.731913	−1.513734
TS33			
0	2	-	-
C	1.224226	0.414494	−0.012889
C	1.558929	−0.529146	1.023592
C	2.799645	−1.100443	1.128883
C	3.813450	−0.675748	0.283850
C	3.600777	0.336844	−0.645840
C	2.360443	0.902137	−0.756166
Cl	0.344053	−1.034509	2.113202
H	2.987315	−1.853789	1.874882
C	−1.348093	−0.517555	−0.792590
C	−2.451062	−1.321314	−1.038641
C	−3.677090	−0.965722	−0.510184
C	−3.826955	0.157166	0.278355
C	−2.712787	0.933562	0.539338
C	−1.472615	0.614305	0.013258
H	−2.357293	−2.210187	−1.639825
H	4.408354	0.683631	−1.267839
H	−4.784230	0.422702	0.692831
S	0.199111	−0.870791	−1.495448
S	−0.067956	1.611871	0.311552
Cl	5.368182	−1.381696	0.411231
Cl	−5.051425	−1.957438	−0.830350
Cl	2.134074	2.135898	−1.916484
Cl	−2.886993	2.332385	1.529253
TS34			
0	2	-	-
C	−1.330092	0.693159	0.131404
C	−2.514557	1.156067	−0.438774
C	−3.660129	0.391680	−0.466982
C	−3.649976	−0.883820	0.108887

Table S3. *Cont.*

TS	*x*	*y*	*z*
TS34			
C	−2.539050	−1.376900	0.716392
C	−1.326071	−0.629201	0.686363
H	−4.554566	0.775792	−0.926668
C	1.304135	−0.723921	−0.596373
C	2.445199	−1.507009	−0.741932
C	3.684549	−0.992097	−0.432664
C	3.817810	0.288809	0.065631
C	2.681144	1.048456	0.244269
C	1.412287	0.576589	−0.088083
H	2.352389	−2.520782	−1.094928
H	−2.539538	−2.337211	1.202563
Cl	−0.282883	−0.942475	2.044681
H	4.783959	0.689799	0.319764
S	0.080749	1.704277	0.102076
S	−0.207723	−1.449823	−1.063357
Cl	−5.100689	−1.807820	0.083610
Cl	5.088584	−1.968039	−0.641290
Cl	−2.573765	2.726098	−1.135843
Cl	2.870980	2.643429	0.869487
TS35			
0	1	-	-
C	−0.753570	−1.150177	1.387177
C	−1.986791	−1.141240	0.684922
C	−2.524588	0.024221	0.248554
C	−1.854185	1.282549	0.455593
C	−0.514469	1.177487	0.998744
C	−0.046820	−0.005460	1.590840
H	−2.506871	−2.071793	0.526382
H	−0.025079	2.113384	1.221759
Cl	−4.042084	−0.005943	−0.541492
H	2.506962	−2.071720	−0.526097
C	1.986842	−1.141214	−0.684782
C	0.753672	−1.150289	−1.387116
C	2.524572	0.024332	−0.248562
C	0.046891	−0.005625	−1.590971
C	1.854089	1.282597	−0.455718
Cl	4.042063	−0.005651	0.541502
C	0.514412	1.177407	−0.998965
H	0.024987	2.113241	−1.222150
H	0.885344	−0.014133	2.131360
H	−0.885200	−0.014409	−2.131612
S	−2.504740	2.751673	0.100646

Table S3. *Cont.*

TS	*x*	*y*	*z*
TS35			
S	2.504468	2.751780	−0.100672
Cl	0.202649	−2.651871	−2.007422
Cl	−0.202399	−2.651673	2.007557
TS36			
0	2	-	-
C	−3.005195	0.621867	−0.960746
C	−3.368499	−0.363048	0.006650
C	−2.436671	−1.137736	0.607368
C	−1.034313	−1.026426	0.293015
C	−0.732401	−0.236333	−0.943006
C	−1.740003	0.745764	−1.373721
H	−4.403799	−0.454693	0.292725
H	−0.811185	−1.060161	−1.761055
Cl	−2.934241	−2.229450	1.830979
H	4.403056	0.382208	0.210420
C	3.364114	0.322853	−0.071403
C	2.953367	−0.709779	−0.971572
C	2.475300	1.180534	0.474598
C	1.685252	−0.809122	−1.367951
C	1.066291	1.129917	0.164596
Cl	3.034500	2.325761	1.621919
C	0.706978	0.254801	−1.016369
H	0.746483	0.951431	−1.867711
H	1.370081	−1.596242	−2.035022
H	−1.461229	1.492005	−2.100622
S	−0.064630	2.015321	0.930339
S	0.151383	−1.773216	1.126942
Cl	−4.245260	1.673724	−1.526275
Cl	4.147093	−1.853107	−1.458280
H	−0.872335	−2.015752	−2.665795
TS37			
0	2	-	-
C	1.468428	−0.033308	1.967194
C	2.770839	0.154790	1.405711
C	2.968970	0.130277	0.070614
C	1.885164	−0.069111	−0.865684
C	0.594154	−0.524204	−0.249291
C	0.416804	−0.318317	1.194125
H	3.600690	0.340172	2.068034
H	0.737566	−1.665074	−0.363663
Cl	4.548561	0.416983	−0.525563
H	−3.731260	1.669833	0.517772

Table S3. *Cont.*

TS	*x*	*y*	*z*
TS37			
C	−2.913128	1.140007	0.056706
C	−1.852037	1.899332	−0.542856
C	−2.920064	−0.208285	0.031864
C	−0.803031	1.297023	−1.096408
C	−1.860910	−0.966353	−0.593507
Cl	−4.247969	−1.036666	0.731958
C	−0.665615	−0.182105	−1.077723
H	−0.439842	−0.515039	−2.091944
O	1.237775	−3.080351	−0.766959
H	0.428808	−3.463817	−1.135661
H	−0.003767	1.860622	−1.550509
H	−0.559133	−0.478731	1.625439
S	−1.930619	−2.585301	−0.761546
S	2.072950	0.081686	−2.479214
Cl	1.317056	0.125204	3.673330
Cl	−2.014707	3.614139	−0.520084
TS38			
0	2	-	-
C	1.350718	0.136391	1.979468
C	2.652524	0.394921	1.458615
C	2.865169	0.484642	0.124657
C	1.796587	0.332654	−0.835888
C	0.538120	−0.242543	−0.274069
C	0.320244	−0.130589	1.164026
H	3.469211	0.550903	2.144518
H	0.790926	−1.419718	−0.424668
Cl	4.440743	0.863028	−0.427297
H	−3.907444	1.428751	0.653487
C	−3.064654	1.003295	0.132813
C	−2.115996	1.890828	−0.485947
C	−2.934865	−0.334242	0.044849
C	−1.030788	1.429221	−1.100372
C	−1.841045	−0.950303	−0.678962
Cl	−4.128254	−1.331934	0.764519
C	−0.729198	−0.028464	−1.128030
H	−0.453618	−0.309377	−2.143896
H	−0.317966	2.092946	−1.562510
H	−0.655865	−0.352650	1.565127
S	−1.763243	−2.540739	−0.987522
S	1.987035	0.644685	−2.429172
Cl	1.157018	0.176860	3.685899
Cl	−2.465146	3.576080	−0.400963
S	1.557562	−3.055475	−0.593351

Table S3. *Cont.*

TS	*x*	*y*	*z*
TS38			
H	2.658037	−2.509804	−0.054989
TS39			
0	2	-	-
C	−2.765781	1.339128	−0.735750
C	−3.452112	0.412326	0.072624
C	−2.800785	−0.658926	0.610399
C	−1.426043	−0.894786	0.373733
C	−0.713273	0.081576	−0.441758
C	−1.439568	1.206634	−0.960351
H	−4.507004	0.540834	0.252935
H	−0.407284	−0.827855	−1.333744
Cl	−3.689110	−1.762721	1.580023
H	4.499008	0.783157	0.463513
C	3.443859	0.655201	0.289673
C	2.543354	1.628083	0.684158
C	2.975767	−0.486732	−0.331128
C	1.183749	1.484692	0.472474
C	1.616678	−0.658185	−0.565305
Cl	4.099971	−1.688040	−0.822175
C	0.728930	0.338110	−0.148724
H	−0.903202	1.941761	−1.539222
H	0.498051	2.248713	0.800993
S	−0.669629	−2.330898	0.797660
S	0.878996	−2.023127	−1.327773
Cl	3.132212	3.047593	1.464274
Cl	−3.654727	2.655818	−1.403472
TS40			
0	2	-	-
C	2.481068	−1.233615	1.102245
C	3.382616	−0.279894	0.587806
C	2.934441	0.784104	−0.132254
C	1.539357	1.017825	−0.426899
C	0.671684	−0.065258	0.003749
C	1.156876	−1.110830	0.854178
H	4.437888	−0.387198	0.780845
H	1.082071	−0.658637	−1.242765
Cl	4.099003	1.916745	−0.683829
H	−4.614740	0.692434	0.548472
C	−3.561431	0.524484	0.402245
C	−2.631821	1.492716	0.764620
C	−3.110941	−0.649622	−0.135530
C	−1.279208	1.299292	0.582705

Table S3. *Cont.*

TS	*x*	*y*	*z*
TS40			
C	−1.723252	−0.896926	−0.360191
C	−0.803418	0.124959	0.030263
H	−0.583449	2.067068	0.874007
H	0.457551	−1.820645	1.266232
O	1.323434	−1.281326	−2.236088
H	2.204596	−1.661353	−2.196626
H	0.642486	−1.976635	−2.118323
S	−1.260623	−2.360000	−1.084559
S	1.024516	2.315803	−1.332420
Cl	−4.288598	−1.823429	−0.553921
Cl	−3.189381	2.947418	1.474268
Cl	3.098271	−2.531363	2.058241
TS41			
0	2	-	-
C	3.121311	1.186013	0.402403
C	3.524230	0.031595	−0.242861
C	2.574141	−0.877878	−0.673433
C	1.221032	−0.651010	−0.464631
C	0.847701	0.532870	0.190240
C	1.782006	1.452225	0.623143
H	4.570218	−0.163808	−0.410803
H	0.448168	−1.190880	1.987727
Cl	3.085914	−2.299471	−1.493953
H	−4.391158	0.404852	−0.490451
C	−3.338610	0.469017	−0.270774
C	−2.647136	1.653548	−0.488405
C	−2.682008	−0.611594	0.257817
C	−1.276440	1.738439	−0.253455
C	−1.287260	−0.574721	0.570159
Cl	−3.564708	−2.045221	0.574837
C	−0.605585	0.659960	0.253338
H	1.478155	2.351298	1.134733
H	−0.735030	2.627070	−0.535953
S	−0.061257	−1.708185	−0.979838
S	−0.859067	−1.456126	2.064943
Cl	−3.483942	3.011117	−1.114918
Cl	4.313001	2.307085	0.947671
TS42			
0	2	-	-
C	3.116646	1.371628	−0.009900
C	3.589355	0.074564	−0.162873
C	2.688973	−0.966776	−0.251482

Table S3. *Cont.*

TS	*x*	*y*	*z*
TS42			
C	1.329008	−0.717119	−0.187418
C	0.870021	0.594743	−0.034422
C	1.769600	1.649409	0.052929
H	4.647758	−0.118797	−0.210819
H	−2.035917	0.200631	2.331776
Cl	3.252518	−2.577958	−0.452188
H	−4.351741	0.173532	−0.656110
C	−3.302961	0.284000	−0.437982
C	−2.743111	1.562281	−0.302062
C	−2.505199	−0.813984	−0.311760
C	−1.382092	1.753788	−0.137401
C	−1.137296	−0.671274	0.101949
Cl	−3.136300	−2.387779	−0.513850
C	−0.565785	0.649552	−0.029359
H	1.430306	2.664675	0.177233
H	−0.974720	2.751437	−0.150521
S	−1.269652	−0.893069	2.237701
S	0.071142	−1.910516	−0.308962
Cl	4.256322	2.660840	0.105742
Cl	−3.778569	2.926133	−0.439274
TS43			
0	1	-	-
C	−1.250840	1.132565	0.935699
C	−2.585340	1.322083	0.980610
C	−3.461200	0.468230	0.267834
C	−2.988091	−0.537649	−0.518111
C	−1.593968	−0.764370	−0.664300
C	−0.722283	−0.004021	0.223349
H	−4.524579	0.632977	0.336737
C	0.722286	−0.004033	−0.223379
C	1.593958	−0.764374	0.664292
C	1.250861	1.132546	−0.935731
C	2.988084	−0.537664	0.518123
C	2.585362	1.322058	−0.980622
H	0.579068	1.771200	−1.486811
C	3.461208	0.468206	−0.267825
H	4.524589	0.632949	−0.336715
H	0.544596	−0.876069	−1.085652
H	−0.579035	1.771221	1.486762
Cl	−4.097406	−1.496198	−1.407450
Cl	4.097380	−1.496210	1.407488
H	−0.544590	−0.876065	1.085639

Table S3. *Cont.*

TS	*x*	*y*	*z*
TS43			
S	−0.892237	−1.879276	−1.678762
S	0.892201	−1.879258	1.678758
Cl	3.277155	2.604388	−1.900981
Cl	−3.277111	2.604424	1.900971
TS44			
0	1	-	-
C	1.837431	−1.225206	1.470394
C	3.015228	−0.574946	1.052423
C	2.988971	0.341361	0.045211
C	1.781205	0.721044	−0.638590
C	0.637899	−0.091552	−0.280301
C	0.663237	−0.961465	0.849929
H	3.948238	−0.799096	1.543765
H	1.102564	−0.977187	−1.273261
Cl	4.461227	1.113491	−0.376391
H	−4.117648	0.963586	1.105995
C	−3.187710	0.798431	0.584965
C	−2.174441	1.801481	0.654790
C	−3.020017	−0.344038	−0.122074
C	−1.019167	1.639531	0.010018
C	−1.805715	−0.631906	−0.829818
C	−0.726554	0.411114	−0.765968
H	−0.242897	2.389838	0.011859
H	−0.243349	−1.458748	1.159994
O	1.289395	−1.743865	−2.209032
H	1.844473	−2.483213	−1.952383
H	0.349428	−2.047413	−2.298884
H	−0.538038	0.761282	−1.794740
Cl	−4.312133	−1.474058	−0.151615
S	−1.652759	−2.031500	−1.688713
S	1.724688	1.895171	−1.820745
Cl	1.928997	−2.351144	2.774653
Cl	−2.519915	3.210537	1.584845
TS45			
0	2	-	-
C	−2.742960	1.369352	−0.849813
C	−3.507134	0.443746	−0.171192
C	−2.872804	−0.548715	0.552617
C	−1.481847	−0.626249	0.636897
C	−0.731804	0.328382	−0.065079
C	−1.363463	1.314081	−0.806672
H	−4.582719	0.481870	−0.208517

Table S3. *Cont.*

TS	*x*	*y*	*z*
TS45			
H	−0.396714	−1.366347	−1.732735
Cl	−3.869255	−1.694217	1.362665
H	4.594911	0.472367	0.273807
C	3.521182	0.443826	0.196260
C	2.735420	1.354322	0.870656
C	2.908569	−0.508754	−0.592717
C	1.360055	1.305245	0.770031
C	1.524145	−0.583993	−0.724210
Cl	3.905830	−1.630729	−1.437415
C	0.749965	0.334411	−0.007770
H	−1.432362	−1.794404	2.687646
H	−2.036829	−1.567310	3.789693
H	0.753238	2.011608	1.312869
H	−0.774141	2.038533	−1.344975
S	0.857308	−1.821312	−1.775423
S	−0.648159	−1.886333	1.532567
Cl	−3.521115	2.605229	−1.761343
Cl	3.480557	2.560428	1.851026
TS46			
0	2	-	-
C	−2.607557	1.754359	−0.554770
C	−3.412871	0.669263	−0.277692
C	−2.827985	−0.532535	0.074475
C	−1.442198	−0.667041	0.179029
C	−0.647995	0.450836	−0.112333
C	−1.231919	1.651604	−0.480846
H	−4.485037	0.748318	−0.340306
H	−0.261836	−0.539881	−2.258685
Cl	−3.871907	−1.859981	0.385340
H	4.656675	0.296260	0.469896
C	3.587605	0.330679	0.344451
C	2.782566	0.952713	1.275474
C	3.001502	−0.246040	−0.764221
C	1.413325	0.987053	1.108125
C	1.624031	−0.225500	−0.969823
Cl	4.022983	−1.009388	−1.921786
C	0.829146	0.390713	0.001950
H	−1.591475	−2.594095	1.542931
H	0.789865	1.464653	1.846574
H	−0.610428	2.503180	−0.705156
S	0.990096	−0.972049	−2.425905
S	−0.657794	−2.173206	0.607122

Table S3. *Cont.*

TS	*x*	*y*	*z*
TS46			
H	−0.972325	−2.136844	3.439568
Cl	−3.329349	3.251600	−1.000047
Cl	3.495762	1.688342	2.661621
O	−1.830685	−2.214234	3.004453
TS47			
0	2	-	-
C	−2.379876	2.138284	0.271564
C	−3.256542	1.135790	−0.088524
C	−2.753913	−0.113141	−0.399310
C	−1.384561	−0.391372	−0.337415
C	−0.517698	0.650296	0.033323
C	−1.017876	1.906513	0.329996
H	−4.316504	1.319711	−0.135046
H	−0.038756	0.810756	−2.329326
Cl	−3.877519	−1.329525	−0.850188
H	4.730792	−0.134427	0.611445
C	3.673525	0.030148	0.489549
C	2.852519	0.210424	1.582083
C	3.119506	0.071400	−0.774656
C	1.497437	0.414786	1.416447
C	1.759588	0.283428	−0.980349
Cl	4.165338	−0.147478	−2.125366
C	0.944363	0.434588	0.145841
H	−1.566567	−2.739654	0.188142
H	0.860175	0.539368	2.277023
H	−0.344449	2.701510	0.606237
S	1.168472	0.329429	−2.632877
S	−0.734916	−1.959030	−0.748347
H	−0.921739	−3.496675	2.145690
S	−2.155572	−3.394711	1.640585
Cl	−2.993337	3.698752	0.651505
Cl	3.524957	0.175990	3.168446
TS48			
0	2	-	-
C	2.462227	−1.995472	0.582114
C	3.310843	−1.029662	0.078461
C	2.776909	0.148635	−0.404047
C	1.396545	0.385565	−0.375704
C	0.554968	−0.619446	0.134576
C	1.091065	−1.801798	0.606897
H	4.375466	−1.189572	0.055975
H	0.091145	−1.242017	−2.145973

Table S3. *Cont.*

TS	*x*	*y*	*z*
TS48			
Cl	3.860603	1.312356	−1.040377
H	−4.707928	0.147450	0.558691
C	−3.647100	−0.015456	0.469554
C	−2.826274	0.036516	1.576873
C	−3.090099	−0.286030	−0.764049
C	−1.466398	−0.164471	1.452744
C	−1.724718	−0.505541	−0.925851
Cl	−4.132435	−0.353621	−2.132608
C	−0.912318	−0.421597	0.209141
H	1.648746	2.751928	−0.429346
H	−0.828685	−0.098240	2.319260
H	0.442199	−2.568775	0.997068
S	−1.124269	−0.855125	−2.538893
S	0.681462	1.853653	−0.958942
Cl	1.746225	3.544244	1.146405
Cl	−3.506129	0.361040	3.124609
Cl	3.120563	−3.461941	1.182292
TS49			
0	1	-	-
C	1.343735	−0.869717	−1.327674
C	2.189822	−0.026072	−0.550169
C	1.791861	1.223996	−0.220902
C	0.510379	1.731525	−0.647724
C	−0.414907	0.727720	−1.153323
C	0.091896	−0.495363	−1.671031
H	3.161352	−0.386647	−0.254491
H	−1.326529	1.126661	−1.578418
Cl	2.856500	2.239530	0.654550
H	0.220450	0.322009	2.008522
C	−0.352747	−0.349121	1.390392
C	−0.045808	−1.655525	1.291639
C	−1.416360	0.187504	0.591544
C	−0.863901	−2.551314	0.536393
C	−2.367745	−0.726224	−0.076092
Cl	−2.060610	1.664752	1.174813
C	−1.972153	−2.106121	−0.089445
H	−2.612959	−2.787968	−0.624561
H	−0.540974	−1.134298	−2.263571
H	−0.589040	−3.593502	0.497203
S	−3.736299	−0.225262	−0.826088
S	0.062549	3.302550	−0.571843
Cl	1.308438	−2.305086	2.127243
Cl	1.984100	−2.383349	−1.830692

Table S3. *Cont.*

TS	*x*	*y*	*z*
TS50			
0	2	-	-
C	−1.278584	1.448915	−1.375257
C	−2.252172	1.220894	−0.336562
C	−2.377200	0.009850	0.234253
C	−1.560828	−1.114077	−0.195692
C	−0.306003	−0.760468	−0.955062
C	−0.377207	0.528579	−1.705365
H	−2.908898	2.026851	−0.052085
H	−0.082577	−1.575301	−1.640442
Cl	−3.589082	−0.239066	1.420509
H	−0.157988	0.236648	1.638191
C	0.764545	0.291858	1.084322
C	1.737298	1.125185	1.459739
C	0.946420	−0.641178	−0.041979
C	2.994542	1.176482	0.769640
C	2.218688	−0.473368	−0.844040
Cl	1.243691	−2.309011	0.811987
C	3.218015	0.404865	−0.309983
H	4.157236	0.451013	−0.836529
H	1.182214	−4.162856	1.462600
H	0.349063	0.706344	−2.481237
H	3.754998	1.848916	1.133534
S	2.473785	−1.279743	−2.242076
S	−1.961765	−2.656358	0.082667
Cl	−1.355358	2.970676	−2.180185
Cl	1.533016	2.173919	2.807833
TS51			
0	2	-	-
C	2.068321	−1.338513	−0.967723
C	2.730658	−0.575530	0.059898
C	2.333779	0.675412	0.353736
C	1.239700	1.309097	−0.364133
C	0.317733	0.375013	−1.111858
C	0.956892	−0.903374	−1.553093
H	3.580215	−1.007753	0.563193
H	−0.076088	0.903217	−1.977222
Cl	3.193770	1.572542	1.533185

Table S3. *Cont.*

TS	*x*	*y*	*z*
TS51			
H	0.145430	−0.001990	1.622892
C	−0.658427	−0.467514	1.077472
C	−1.468672	−1.356919	1.693668
C	−0.905425	−0.059210	−0.282978
C	−2.559989	−1.965019	1.017668
C	−1.958474	−0.792829	−1.029164
Cl	−1.983892	1.667400	0.066165
C	−2.792184	−1.679072	−0.285331
H	−3.608250	−2.143705	−0.814088
O	−2.543715	3.653096	−0.129335
H	−1.745330	3.948016	−0.585389
H	0.459235	−1.479298	−2.315474
H	−3.190792	−2.654470	1.554903
S	1.026892	2.913781	−0.387803
S	−2.205321	−0.582478	−2.648165
Cl	−1.196676	−1.783019	3.333135
Cl	2.799544	−2.834505	−1.408228
TS52			
0	2	-	-
C	2.580860	−0.146030	−1.093200
C	2.834559	0.796642	−0.032901
C	1.886510	1.666761	0.357194
C	0.586111	1.705426	−0.290703
C	0.206572	0.470590	−1.072776
C	1.368490	−0.297059	−1.619102
H	3.813785	0.822309	0.416939
H	−0.451274	0.767280	−1.886639
Cl	2.253757	2.813837	1.576328
H	0.487774	−0.139763	1.608986
C	−0.034423	−0.899882	1.052124
C	−0.241586	−2.117106	1.591524
C	−0.587739	−0.558050	−0.244314
C	−0.960590	−3.131666	0.895726
C	−1.190087	−1.670049	−1.038471
Cl	−2.296060	0.359135	0.340411
C	−1.417735	−2.904746	−0.355264
H	−1.944272	−3.673107	−0.897405
H	−3.451728	2.767755	−0.450382
H	1.179950	−1.006759	−2.407479
H	−1.123891	−4.080450	1.380789
S	−0.391891	2.992109	−0.214354

Table S3. *Cont.*

TS	*x*	*y*	*z*
TS52			
S	−1.628921	−1.493164	−2.611633
Cl	0.358795	−2.489746	3.155796
Cl	3.931502	−1.056306	−1.654019
S	−4.286181	1.838313	0.027325
TS53			
0	2	-	-
C	2.631245	−0.210239	−0.979439
C	2.836029	0.766572	0.062128
C	1.878624	1.660745	0.363933
C	0.620110	1.692222	−0.365859
C	0.269097	0.430206	−1.117484
C	1.450092	−0.369685	−1.570594
H	3.788253	0.796627	0.566248
H	−0.342425	0.703933	−1.974057
Cl	2.183333	2.849810	1.558251
H	0.413265	−0.100242	1.594395
C	−0.095528	−0.867759	1.035168
C	−0.350812	−2.068168	1.603853
C	−0.570929	−0.558382	−0.294138
C	−1.049895	−3.090998	0.906700
C	−1.173006	−1.680342	−1.073619
Cl	−2.283987	0.393676	0.249864
C	−1.448390	−2.891557	−0.371401
H	−1.965316	−3.666023	−0.914014
Cl	−4.163184	1.917118	0.056130
H	1.299720	−1.103933	−2.345170
H	−1.249828	−4.023231	1.409651
S	−0.349739	2.981529	−0.414439
S	−1.548802	−1.540629	−2.668860
Cl	0.171069	−2.394481	3.202406
Cl	4.001779	−1.150666	−1.431342
TS54			
0	2	-	-
C	2.079208	1.732539	0.517600
C	2.908308	0.897435	−0.256012
C	2.483894	−0.341536	−0.638620
C	1.213431	−0.844287	−0.272472
C	0.347132	0.035540	0.501846
C	0.831725	1.337997	0.859276
H	3.894592	1.233043	−0.532331
H	0.298991	−0.821780	1.497545
Cl	3.541833	−1.322302	−1.570615

Table S3. *Cont.*

TS	*x*	*y*	*z*
TS54			
H	−1.397927	1.742973	−0.829635
C	−1.872066	0.885339	−0.379538
C	−3.239238	0.706876	−0.491336
C	−1.130148	−0.061227	0.302825
C	−3.870464	−0.395361	0.062110
C	−1.755715	−1.171924	0.871819
C	−3.126636	−1.340623	0.740308
H	−3.609923	−2.202980	1.170205
H	0.177712	1.996126	1.409177
H	−4.937217	−0.508690	−0.042447
S	0.776408	−2.447860	−0.504129
S	−0.684966	−2.254166	1.701709
Cl	−4.174740	1.879029	−1.345051
Cl	2.685502	3.275573	0.989747
TS55			
0	2	-	-
C	−3.074869	−0.323617	−0.902829
C	−3.715416	0.789723	−0.308917
C	−3.000189	1.691763	0.408941
C	−1.585567	1.589343	0.624434
C	−0.984631	0.368597	0.122363
C	−1.746815	−0.505237	−0.729234
H	−4.776716	0.923836	−0.451459
H	−1.443717	−0.172086	1.373707
H	4.300399	−0.112401	−0.663856
C	3.244988	−0.033365	−0.466666
C	2.574035	1.168083	−0.664717
C	2.536318	−1.115582	−0.022947
C	1.222332	1.290907	−0.423187
C	1.142667	−1.038959	0.271502
C	0.487627	0.209873	0.028795
H	0.729392	2.232401	−0.593254
H	−1.246759	−1.337063	−1.200057
O	−1.765721	−0.793270	2.346818
H	−2.714849	−0.940272	2.345198
H	−1.290093	−1.622572	2.124268
S	0.370279	−2.401981	0.923494
S	−0.803473	2.740446	1.550839
Cl	3.399675	−2.581488	0.195350
Cl	3.452894	2.514545	−1.253828
Cl	−4.006452	−1.412274	−1.868217
H	−3.487660	2.556389	0.830178

Table S3. *Cont.*

TS	*x*	*y*	*z*
TS56			
0	2	-	-
C	2.523806	1.555582	0.248102
C	3.142462	0.450015	−0.305030
C	2.389903	−0.669938	−0.612413
C	1.023375	−0.699694	−0.373764
C	0.427549	0.439872	0.187058
C	1.163378	1.565674	0.497319
H	4.202457	0.455015	−0.496673
H	0.462676	−1.630274	2.013375
Cl	3.165433	−2.032794	−1.319537
H	−1.576118	2.118583	−0.600700
C	−1.913817	1.159910	−0.239670
C	−3.247550	0.790715	−0.395571
C	−1.016571	0.276425	0.299329
C	−3.680928	−0.489681	−0.055718
C	−1.435722	−1.040887	0.718320
C	−2.804715	−1.380491	0.500312
H	−3.149141	−2.352241	0.817220
H	0.693297	2.429916	0.938188
H	−4.713220	−0.760015	−0.207819
S	−0.845410	−1.716014	2.269708
S	−0.031208	−2.034189	−0.748367
Cl	−4.361003	1.912697	−1.061125
Cl	3.470801	2.943465	0.638622
TS57			
0	2	-	-
C	2.529535	1.655684	0.107467
C	3.250788	0.489660	−0.112983
C	2.573640	−0.698327	−0.297708
C	1.190577	−0.723141	−0.262317
C	0.478883	0.460752	−0.041672
C	1.153032	1.660883	0.142990
H	4.327237	0.508931	−0.137942
H	−2.367933	−0.703371	2.198459
Cl	3.446509	−2.151674	−0.583890
H	−1.746486	2.223031	−0.097265
C	−1.953845	1.166679	−0.155115
C	−3.247711	0.729111	−0.374399
C	−0.939413	0.235569	−0.082639
C	−3.543543	−0.627409	−0.596287
C	−1.253122	−1.174267	−0.052228
C	−2.561605	−1.565167	−0.499417

Table S3. *Cont.*

TS	*x*	*y*	*z*
TS57			
H	−2.774810	−2.608551	−0.664693
H	0.618687	2.579824	0.320156
H	−4.550625	−0.911009	−0.853897
S	−1.373835	−1.586958	2.046755
S	0.188553	−2.122220	−0.502037
Cl	−4.521156	1.879619	−0.473959
Cl	3.392008	3.130751	0.344057
